# Membrane-induced tau amyloid fibrils

**DOI:** 10.1038/s42003-023-04847-6

**Published:** 2023-04-28

**Authors:** Nadia El Mammeri, Olivia Gampp, Pu Duan, Mei Hong

**Affiliations:** Department of Chemistry, Massachusetts Institute of Technology, Cambridge, MA USA

**Keywords:** Solid-state NMR, Membrane structure and assembly

## Abstract

The intrinsically disordered protein tau aggregates into β-sheet amyloid fibrils that spread in human brains afflicted with Alzheimer’s disease and other neurodegenerative diseases. Tau interaction with lipid membranes might play a role in the formation and spreading of these pathological aggregates. Here we investigate the conformation and assembly of membrane-induced tau aggregates using solid-state NMR and transmission electron microscopy. A tau construct that encompasses the microtubule-binding repeats and a proline-rich domain is reconstituted into cholesterol-containing phospholipid membranes. 2D ^13^C-^13^C correlation spectra indicate that tau converted from a random coil to a β-sheet conformation over weeks. Small unilamellar vesicles (SUVs) cause different equilibrium conformations from large unilamellar vesicles (LUVs) and multilamellar vesicles (MLVs). Importantly, SUV-bound tau developed long fibrils that exhibit the characteristic β-sheet chemical shifts of Tyr310 in heparin-fibrillized tau. In comparison, LUVs and MLVs do not induce fibrils but cause different β-sheet aggregates. Lipid-protein correlation spectra indicate that these tau aggregates reside at the membrane-water interface, without inserting into the middle of the lipid bilayer. Removal of cholesterol from the SUVs abolished the fibrils, indicating that both membrane curvature and cholesterol are required for tau fibril formation. These results have implications for how lipid membranes might nucleate tau aggregates.

## Abstract (web summary)

Solid-state NMR and TEM reveal time-dependent changes in the conformation, dynamics, and morphology of the tau protein upon interactions with lipid membranes, such as SUVs, LUVs, and MLVs with different lipid compositions.

## Introduction

Tau is an intrinsically disordered and highly cationic protein that associates with and stabilizes microtubules in neurons^1^. However, in human brains afflicted with Alzheimer’s disease (AD), abnormal hyperphosphorylation^2^ and other post-translational modifications^3^ of tau perturb the delicate electrostatic balance, causing tau to dissociate from microtubules and subsequently self-assemble into pathological amyloid fibrils^4,5^. The spread of tau aggregates in AD brains follows a characteristic spatiotemporal pattern, which forms the basis of neuropathological staging of AD^6^. Elucidating the molecular details of tau aggregation in human brains is therefore essential for the diagnosis, treatment and prevention of AD. To dissect the evolution of tau from its functional state to its dysfunctional state, it is necessary to investigate the molecular details of how tau binds microtubules, interacts with lipid membranes, forms intermediate oligomeric species, and assembles into β-sheet rich amyloid fibrils. Among these states of tau, the best known so far is the amyloid fibril found in AD brains. In addition to AD, a number of other neurodegenerative diseases, such as Pick’s disease, chronic traumatic encephalopathy, corticobasal degeneration and progressive supranuclear palsy^7–11^, are also characterized by abnormal tau aggregates. In addition to fibrils, microtubule-bound tau has also been studied in detail using cryoelectron microscopy (cryoEM)^12^, solution NMR^13–17^ and solid-state NMR^18^. These studies have provided information about the microtubule-bound conformation of the cationic repeats in full-length tau and the mobility of the flanking regions.

Compared to the fibrillar and microtubule-bound states of tau, the conformation and dynamics of membrane-bound tau is less understood. Interest in deciphering tau interactions with lipid membranes is motivated by the observation that the lipid membrane is physically associated with tau filaments in diseased brains. Electron micrographs of the frontal cerebral cortex of patients with advanced AD showed paired helical filament (PHF) tau stemming from the endoplasmic reticulum membrane^19^. HPLC analysis of AD PHF tau found tightly associated phosphatidylcholine, sphingomyelin, and cholesterol^20^. Lipid rafts in a mice model of AD show enrichment of phosphorylated tau over time^21^. In addition to in vivo data, fluorescence spectroscopy, microscopy and other biophysical data have shown that various tau constructs interact avidly with negatively charged lipids in vitro^22–25^. Recently, biochemical evidence emerged that tau accumulates in synapses prior to AD pathology^26,27^, suggesting that tau might interact with synaptic vesicles and cross the membrane to propagate from one neuron to another.

On a fundamental level, the lipid membrane can potentially play three roles in tau aggregation. First, the two-dimensional nature of the membrane can concentrate tau to promote its aggregation. Second, the negatively charged membrane surface may have favorable electrostatic interactions with cationic residues in tau. Third, the membrane represents a barrier that tau species may need to cross in order to spread the pathology^23,28^. Among these factors, the enrichment of negatively charged lipids such as phosphatidylserine in AD brains over healthy brains^29,30^ is noteworthy, because this might favor tau interactions with lipid membranes in a similar way as the induction of tau fibrillization by anionic polymers such as heparin^31^ and RNA^32^. On the other hand, the lipid membrane also differs from the anionic cofactors and from the microtubules by its increased disorder and the intermolecular nature of its negative charges.

**Fig. 1:**
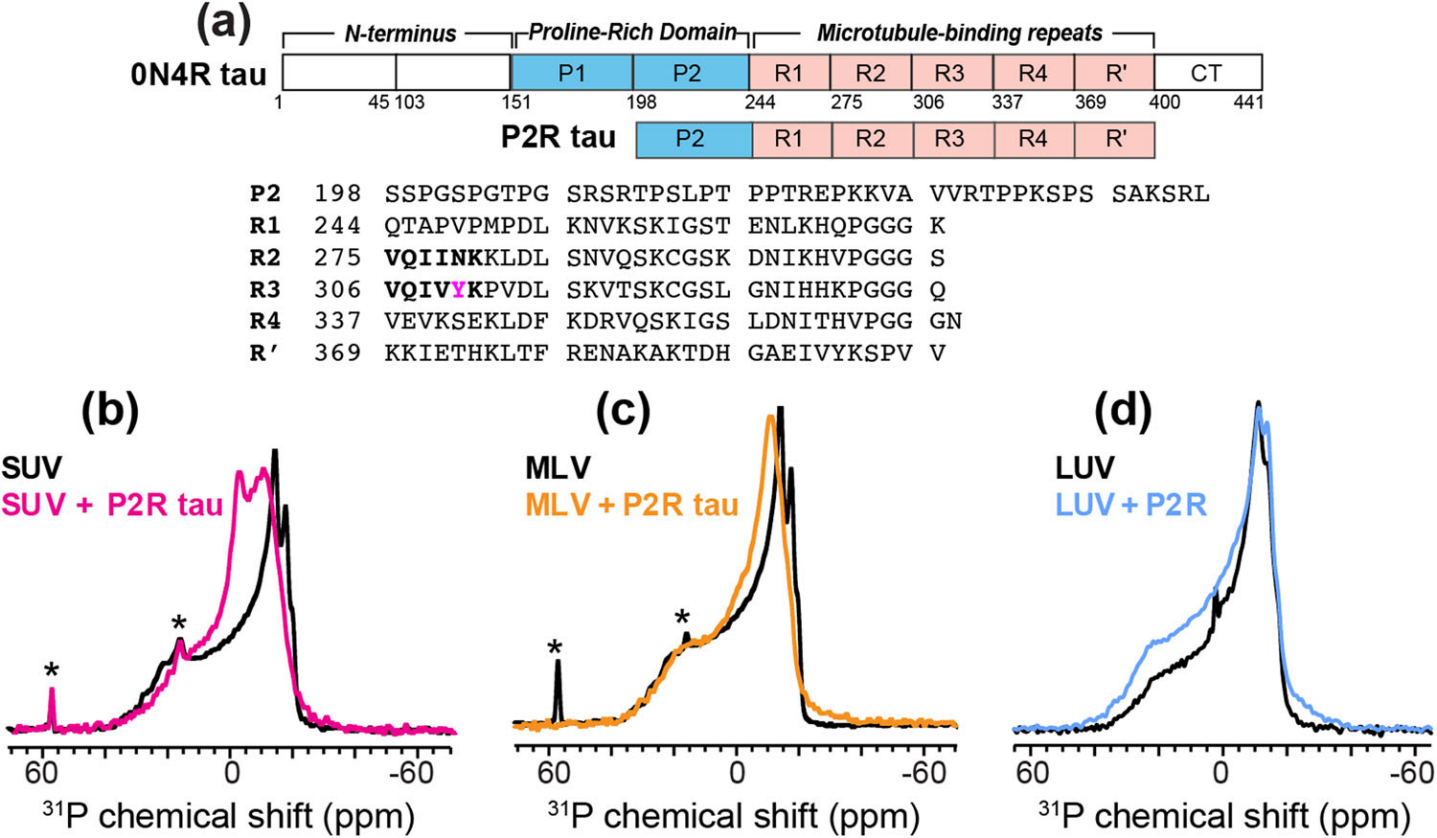
P2R tau binds and increases the mobility of SUV membranes. **a** Amino acid sequence of P2R tau. The sequence diagram of full-length 0N4R tau is shown for comparison. **b** Static ^31^P NMR spectra of freshly prepared SUVs without (black) and with (magenta) P2R tau. Tau binding reduced the ^31^P CSA of the membrane, as shown by the emergence of an isotropic peak. **c** Static ^31^P NMR spectra of freshly prepared MLVs without (black) and with (orange) P2R tau. **d** Static ^31^P NMR spectra of freshly prepared LUVs without (black) and with (cyan) P2R tau. No obvious lineshape changes are observed for the MLV and LUV samples upon tau binding. These ^31^P spectra were measured at a sample temperature of 295 K. All three membranes consist of POPC, POPE, POPS and cholesterol.

To better understand tau interactions with lipid membranes, here we use solid-state NMR and negative-stain transmission electron microscopy (TEM) to chart time-dependent changes in the conformation, dynamics, and morphology of membrane-bound tau. We prepared four different membranes: small unilamellar vesicles (SUVs), large unilamellar vesicles (LUVs), and multilamellar vesicles (MLVs) made of a mixture of zwitterionic lipids, anionic lipids and cholesterol, and SUVs made without cholesterol. These membrane properties were chosen to mimic the high-curvature membranes at the synaptic junction and the low-curvature membrane of axons, and to assess the impact of membrane cholesterol on tau aggregation, as cholesterol level is enhanced in AD brains over healthy brains^33,34^. We use a tau construct that spans residues 198–399, which includes the proline-rich P2 domain, all four microtubule-binding repeats (R1 to R4), and the pseudo-repeat R’ (Fig. 1a). This P2R tau encompasses the β-sheet cores of all brain-derived tau fibrils known so far, moreover the full R’ domain is now known to anchor onto the microtubules^18^. This P2R tau construct resembles the K27 construct^35^, but additionally includes the R2 repeat. We show that P2R tau is disordered and dynamic when freshly bound to lipid membranes, but over the course of days and weeks acquires clear β-sheet content. Importantly, the type of β-sheet conformation differs for the different membranes, with the cholesterol-containing SUVs inducing amyloid fibrils.

## Results

**Fig. 2:**
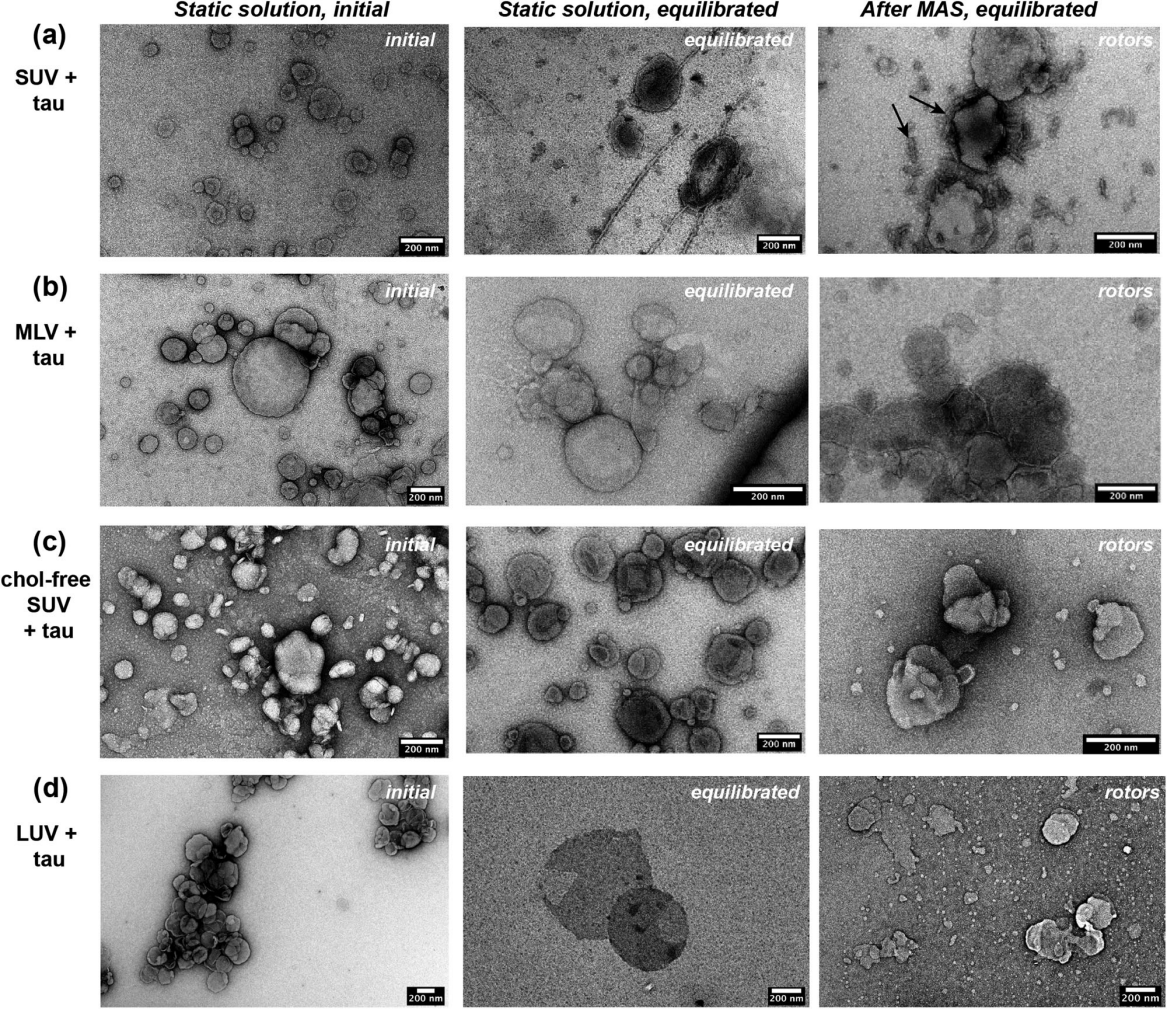
Negative stain TEM images show the initial and equilibrated morphologies of tau-bound lipid vesicles. **a** SUV-bound P2R tau. **b** MLV-bound P2R tau. **c** Cholesterol-free SUV-bound P2R tau. **d** LUV-bound P2R tau. All scale bars are 200 nm. Left column: images of unshaken and freshly prepared membrane vesicle solutions. Middle column: images of unshaken vesicle solutions that have equilibrated for several weeks. Right column: images of samples that have been spun in the MAS rotor for several weeks. Tau-bound SUVs develop long and 15-nm wide fibrils that stem from the vesicles in static solution. After MAS, short fibrils of 50–70 nm long are observed on the surface of the SUVs. Tau-bound MLVs have diameters of 200–600 nm and do not exhibit filaments in the mature state. Tau-bound cholesterol-free SUVs do not show fibrils. In all images, membrane vesicles remain mostly intact. A detailed description of these samples is given in Supplementary Table 4.

To mimic tau interactions with high-curvature membranes at the presynaptic terminal, we produced cholesterol-containing SUVs by sonication. After adding the protein to the SUVs and shaking the mixture at 300 rpm overnight at 37 °C, P2R tau co-sedimented with the SUVs nearly quantitatively, as shown by SDS-PAGE gels (Supplementary Fig. 1). Incubation at room temperature abolished the co-sedimentation, leaving most protein in solution. Therefore, P2R tau has substantial interactions with cholesterol-containing SUVs at physiological temperature. To mimic tau interactions with axonal membranes, we produced low-curvature proteoliposomes by either co-assembling tau and lipids in MLVs or by adding tau to extruded LUVs. TEM images of freshly prepared tau-containing proteoliposomes show that the SUVs have diameters of 30–150 nm, LUVs have diameters of 200–300 nm, whereas MLVs have diameters of 50–300 nm (Fig. 2, Supplementary Fig. 2).

We investigated the impact of tau on the membrane integrity by measuring static ^31^P NMR spectra of the tau-lipid mixtures before they were subject to magic-angle spinning (MAS). The protein-free membranes show a uniaxial ^31^P NMR powder lineshape, which is characteristic of the lamellar morphology of the lipid bilayers (Fig. 1b–d). Two components can be observed in the overlapped powder pattern due to the slightly different chemical shift anisotropies (CSAs) of POPC, POPS and POPE^36^. The largest chemical shift span is 46 ppm for SUVs, 47 ppm for LUVs, and 45 ppm for MLVs, corresponding to the CSA of POPC^37^. Tau binding reduced the largest ^31^P CSA to 39 ppm for the SUV sample, 42 ppm for the MLV sample, and 43 ppm for the LUV sample. Moreover, it caused an isotropic peak in the SUV spectrum. This reduction of ^31^P CSA indicates that tau binding accelerates lipid reorientational motion, which likely results from smaller membrane vesicles so that lipid lateral diffusion leads to faster reorientation^38^. Small vesicles can also undergo isotropic tumbling to average the ^31^P CSA. This isotropic peak is consistent with the presence of many 25–75 nm diameter vesicles in the TEM images of the tau-bound SUVs (Supplementary Fig. 2a).

We next assessed the mobility of membrane-bound P2R tau using ^13^C cross polarization (CP), direct polarization (DP) and INEPT experiments under MAS (Supplementary Fig. 3). The CP and INEPT experiments preferentially detect rigid and highly mobile segments, respectively, while the DP experiment gives approximately quantitative intensities. All samples show much lower CP intensities than the DP and INEPT spectra, indicating that tau is highly dynamic in the freshly prepared proteoliposomes.

**Fig. 3:**
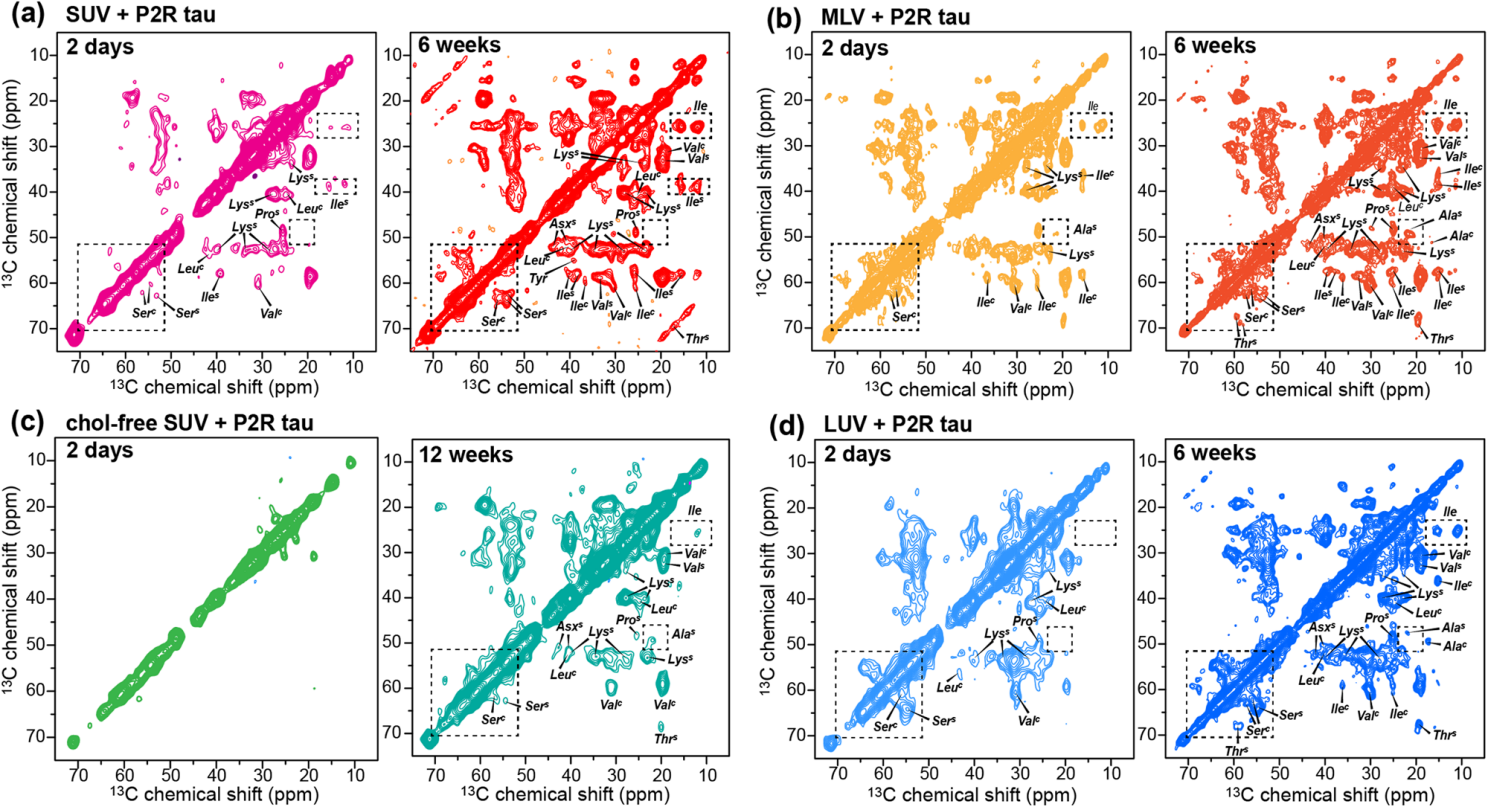
2D ^13^C-^13^C correlation spectra indicate slow conversion of membrane-bound tau from a disordered state to a β-sheet rich conformation. **a** 2D CC spectra of SUV-bound tau after 2 days and after 6 weeks, measured with CORD mixing times of 100 ms and 50 ms, respectively. β-sheet Ser, Val, Ile, and Lys intensities developed over time. A Tyr Cα-Cβ cross peak at (55, 39) ppm is observed. **b** 2D CC spectra of MLV-bound tau measured with 50 ms mixing. β-sheet Ser, Ala, Ile and Lys intensities developed over time. **c** 2D CC spectra of Cholesterol-free SUV-bound tau measured with 100 ms CORD mixing. Almost no cross peaks were present initially. β-sheet signals grew over time but their intensities are weaker than the CHOLESTEROL-containing membrane samples. **d** 2D CC spectra of LUV-bound tau measured with 50 ms mixing. Both random-coil and β-sheet signals of Ser, Ala, and Lys developed over time.

2D ^13^C-^13^C (CC) correlation spectra provided site-resolved information about the conformation and dynamics of membrane-bound tau. In freshly prepared cholesterol-containing SUVs, MLVs and LUVs, tau exhibits mainly random coil Val, Ile and Pro signals (Fig. 3a, b, d). The only β-sheet signals in these fresh membranes are those of Lys residues, indicating that the cationic Lys’s interact with the lipid membrane more avidly than other residues. Interestingly, both the freshly prepared SUV sample and the freshly prepared LUV sample show no Ile methyl sidechain signals, indicating that tau lacks a folded hydrophobic core upon its initial binding to the SUVs and LUVs.

After several weeks, the spectra of all three cholesterol-containing samples changed substantially. Numerous β-sheet cross peaks appeared, indicating that tau slowly converted to β-sheet rich conformations in the cholesterol-containing membranes. But the chemical shift patterns differ among the three membranes. For SUV-bound tau, strong β-sheet Ser, Lys, Val and Ile signals are observed but no β-sheet Ala signal is present. The Ile methyl peak intensities increased dramatically in both the SUV and LUV samples, indicating the development of a tertiary structure in the equilibrated tau-lipid mixture. The equilibrated MLV-bound tau exhibits β-sheet Ser, Ile, Val, and Thr signals, and in addition shows a β-sheet Ala peak. Ala residues occur only in the P2 and R’ domains of the protein. 2D ^15^N-^13^C correlation spectra (*vide infra*) allowed the assignment of the immobilized Ala residues to the R’ domain, indicating that R’ constitutes part of the membrane-immobilized β-sheet core. The MLV-bound tau displays Ile sidechain methyl signals at both the initial and final stages, indicating a well-formed tertiary structure. Finally, the equilibrated LUV sample exhibits a similar 2D CC spectrum as the MLV sample, including the presence of β-sheet Ala peaks, indicating that these two membranes promote similar tau conformation and dynamics.

To investigate whether cholesterol in the membrane affects tau binding and conformation, we also prepared a cholesterol-free SUV sample (Fig. 3c). In contrast to the cholesterol-containing membranes, the fresh cholesterol-free SUV-bound tau lacks any ^13^C-^13^C cross peaks, indicating that the protein is sufficient mobile to average the ^13^C-^13^C dipolar coupling but not sufficiently dynamic to average the stronger ^1^H-^13^C dipolar coupling. This intermediate mobility has been previously observed for the Pro-rich domain of full-length tau in heparin-induced fibrils^39^. Despite the near absence of cross peaks, the protein developed weak β-sheet signals after several weeks, except that the Ile sidechain signals remain largely absent, indicating that cholesterol depletion prevents the formation of a folded hydrophobic core for the protein.

**Fig. 4:**
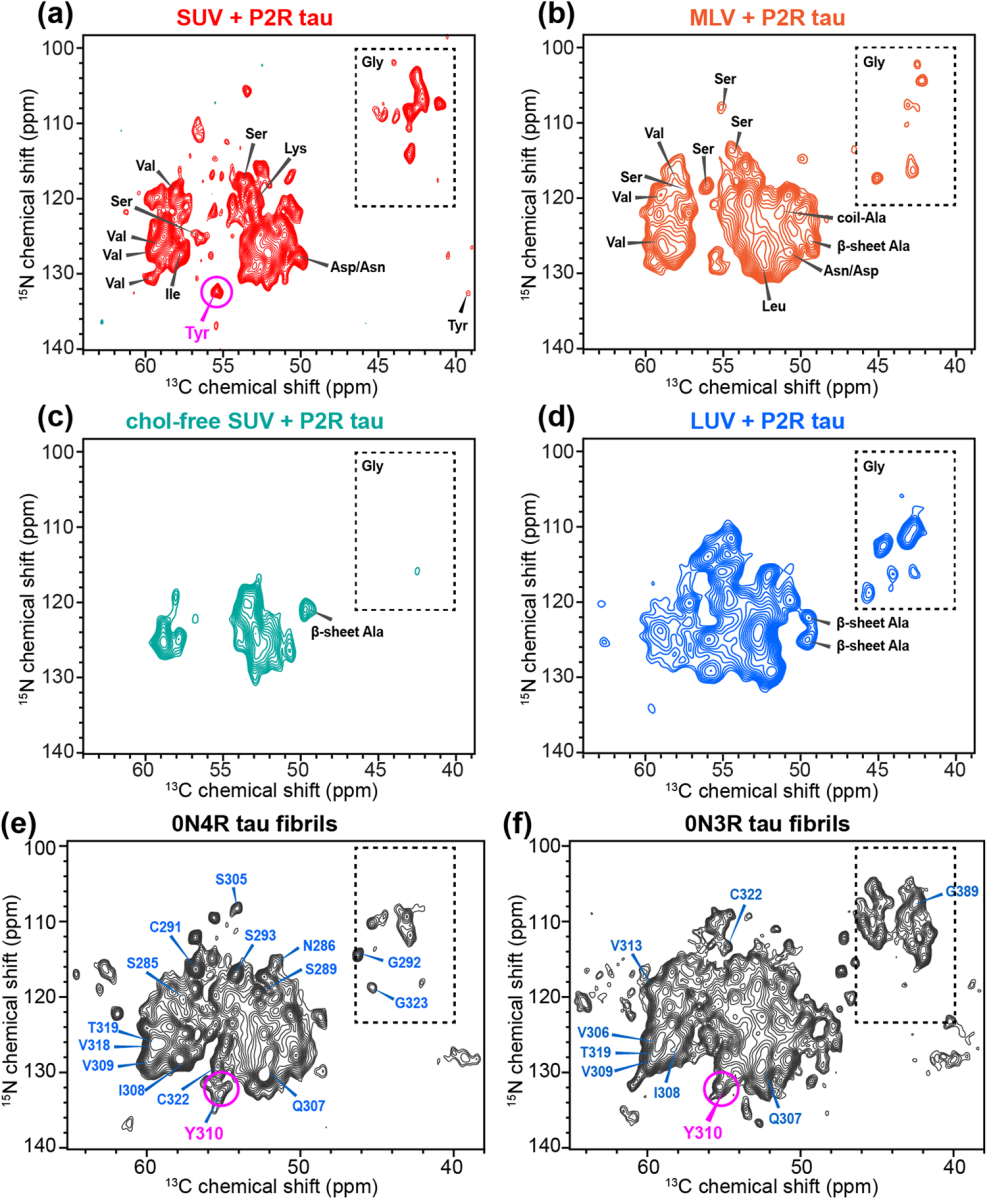
2D NCA correlation spectra indicate that equilibrated SUV-bound tau has an amyloid-fibril like structure, which differs from MLV- and LUV-bound tau. **a** Spectrum of SUV-bound P2R tau. The Tyr/Phe peak that matches the Y310 peak in heparin-fibrillized tau is circled. A weak Cβ chemical shift is also observed. **b** Spectrum of MLV-bound P2R tau, showing broader linewidths, no Tyr/Phe peak, and few Gly peaks compared to the SUV spectrum. **c** Spectrum of Cholesterol-free SUV-bound P2R tau, showing broader linewidths, no Gly nor Tyr/Phe signals compared to the SUV sample. But a β-sheet Ala signal is observed. **d** Spectrum of LUV-bound P2R tau, also showing β-sheet Ala signals. **e** Spectrum of heparin-induced 0N4R tau fibrils^39^. Some of the sequentially assigned peaks are given. **f** Spectrum of heparin-induced 0N3R tau fibrils^41^, showing some of the sequential assignments.

To obtain additional information about the identity of the β-sheet domain, we measured 2D ^15^N-^13^Cα correlation spectra for the four membrane samples (Fig. 4). The SUV-bound tau displays the best resolved spectra among the four samples, with ^15^N linewidths of 0.7–1.3 ppm and ^13^C linewidths of 0.6–1.0 ppm, indicating that SUV-bound tau has the most ordered β-sheet core. Several resolved Gly signals are observed. Together with the Pro signals in the 2D CC spectra (Fig. 3a), these suggest that at least one of the four PGGG motifs is immobilized by the cholesterol-containing SUVs^18^. We combined the 2D NCα spectra with intra-residue 2D N(CA)CX spectra, inter-residue 2D N(CO)CX spectra, and 2D CC spectra to assign the residue types and several sequence-specific residues (Supplementary Fig. 4). 3D correlation experiments for full resonance assignment do not have sufficient sensitivity on these samples due to the large molecular weight of P2R tau (21 kDa) and dilution of the protein by lipids. For SUV-bound tau, we found three sequential connectivities: GSV or GSL, GG and VY. Several GG motifs occur at the C-terminal end of each repeat from R1 to R4. There are two VY doublets in the protein, located in the R3 and R’ repeats (Supplementary Fig. 4b, Supplementary Tables 2 and 3). Importantly, the Tyr residue in the VY pair has ^15^N, Cα and Cβ chemical shifts of 132.2 ppm, 55.4 ppm and 39.0 ppm (Figs. 4a, and 3a, Supplementary Fig. S5), which match the Y310 chemical shifts in heparin-fibrillized full-length 0N4R tau^40^, 0N3R tau^41^ (Fig. 4e, f) and several shorter tau constructs^42–44^. In particular, the large downfield ^15^N chemical shift of 132 ppm is diagnostic of Y310 in tau amyloid fibrils. The observation of these Tyr chemical shifts thus strongly suggests that Y310 in SUV-bound tau adopts a similar conformation as in tau amyloid fibrils formed in solution. Y310 is located in the hexapeptide motif ^306^VQIVYK^311^ of tau (Fig. 1a), which is well known to have a high propensity for forming β-sheet conformation, stabilized by both backbone hydrogen bonds and sidechain interdigitation^45,46^. All tau fibrils known to date^7–9,47^ include this R3 hexapeptide motif in the rigid cores. Therefore, the observation of the characteristic β-sheet steric-zipper Y310 chemical shifts in SUV-bound tau provides strong evidence that cholesterol-containing SUVs induce tau fibrils whose β-sheet core includes the R3 hexapeptide motif. This conclusion is also consistent with the observation of GSV or GSL triplets, which occur only in R3 and R4 repeats.

Compared to the SUV-bound tau, LUV, MLV, and cholesterol-free SUV samples show broader linewidths, increased resonance overlap, and fewer or no Gly signals in the 2D NCα spectra (Fig. 4b–d). Moreover, no (^15^N, ^13^Cα) cross peak at (132 ppm, 56 ppm) is detected in these spectra, strongly suggesting that Y310 does not adopt a steric-zipper β-strand conformation in MLV, LUV, and cholesterol-free SUV membranes (Supplementary Fig. 5). Therefore, these membrane-bound tau samples are less ordered than SUV-bound tau. The lack of an extended β-sheet assembly in the MLV sample is not surprising, since the 2-nm spacing between adjacent bilayers in a multilamellar vesicle is expected to restrict the formation of long fibrils. From the 2D N(CO)CX and N(CA)CX spectra of MLV-bound tau, we assigned a sequential AK motif, which occurs only in the R’ and P2 domains of the protein (Supplementary Fig. 4c, d). 2D ^1^H-^13^C INEPT spectra of the SUV, MLV and LUV samples (Supplementary Fig. 6) show high intensities for Pro-preceding Ala, Ser and Thr peaks and a Met peak, indicating that the P2 and R1 domains are highly mobile. These data thus rule out a rigid P2 domain, and make R’ the only domain that can give rise to the Ala-Lys peaks in the dipolar 2D ^15^N-^13^C correlation spectra.

To validate and obtain additional information about the type of assembly of tau in these membrane environments, we measured negative-stain TEM images of the lipid-tau mixtures after incubating at 37 °C under the static condition for several weeks. Remarkably, SUV-bound tau developed fibrils that are 14–18 nm wide and up to 1 μm long (Fig. 2a). In contrast, the co-assembled MLVs, LUVs and cholesterol-free SUVs did not develop fibrils (Fig. 2b–d). Amyloid fibril induction by the static SUV solution is in excellent agreement with the observation of the amyloid β-sheet Y310 chemical shifts, whereas the absence of fibrils in the other membranes is consistent with the lack of these chemical shifts. After the proteoliposomes had been subject to spinning for many weeks, we examined the morphologies of the samples from the rotors. Despite the highly concentrated nature of these samples and the history of spinning, the morphological differences among these MAS samples are consistent with the morphological differences among the dilute static solutions. The SUV-bound tau displays 12–15 nm wide and 50–70 nm long stubs of fibrils, most of which on the SUV surface. In contrast, no fibrils were observed in the MLV, LUV and cholesterol-free SUV rotors. The short length of the SUV-bound tau fibrils in the MAS rotors is expected, as magic-angle spinning exerts strong centrifugal and shearing force to the samples.

**Fig. 5:**
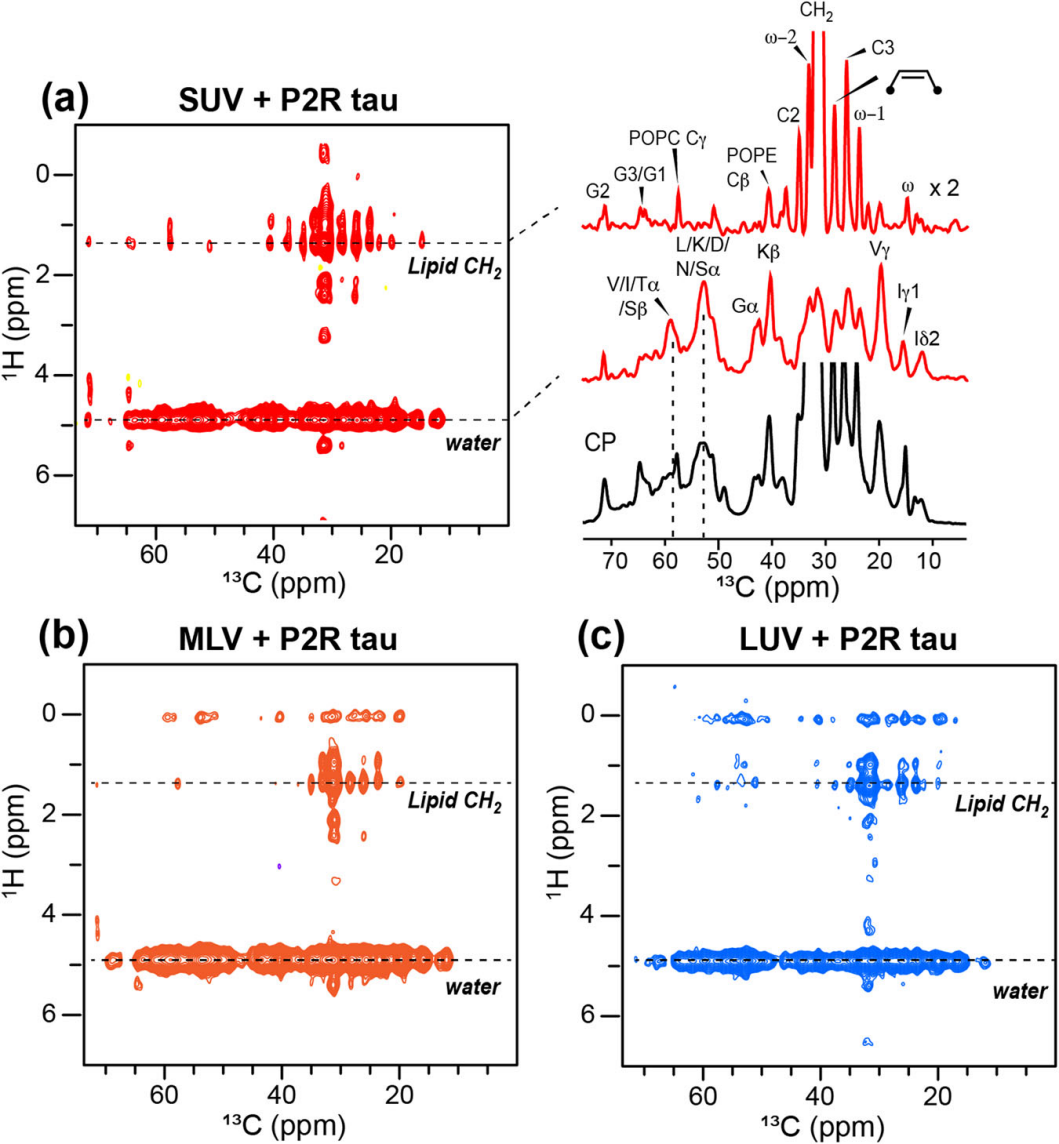
2D ^1^H-^13^C HETCOR spectra indicate that tau does not insert into lipid membranes under the current experimental conditions. **a** 2D ^1^H-^13^C HETCOR spectrum of SUV-bound P2R tau. ^13^C cross sections at the lipid CH_2_ and water ^1^H chemical shifts are shown on the right. Key protein and lipid ^13^C signals are assigned. No protein ^13^C to lipid acyl chain ^1^H correlations are observed, indicating that tau does not insert into the hydrophobic interior of the membrane. **b** 2D ^1^H-^13^C HETCOR spectrum of MLV-bound P2R tau. **c** 2D ^1^H-^13^C HETCOR spectrum of LUV-bound P2R tau. No protein-lipid cross peaks are observed in any of these membranes. The three spectra were measured using a ^1^H mixing time of 100 ms after a ^1^H T_2_ filter of 2 × 2.18 ms to remove the rigid protein signals.

To investigate whether the β-sheet tau assemblies insert into lipid bilayers, we measured 2D ^1^H-^13^C spectra that correlate the ^1^H signals of water and lipid chains with the protein ^13^C signals. Even with a long ^1^H mixing time of 100 ms^48^, the spectra displayed only water-protein cross peaks but no lipid-protein cross peaks (Fig. 5). All cross peaks in the lipid ^1^H cross section result from intramolecular lipid cross peaks. This indicates that tau is associated with the membrane–water interface, without inserting into the hydrophobic interior of the lipid bilayers.

To compare the mobilities of the four membrane-bound tau samples, we measured ^13^C-^1^H dipolar couplings using the 2D DIPSHIFT experiment (Supplementary Fig. 7). The experiment was conducted using ^1^H-^13^C CP contact times of 70 μs and 500 μs to probe the dynamics of the most rigid residues versus both rigid and semi-rigid residues. The experiment was carried out at −2 °C and 15 °C, to assess if the protein mobility depends on the membrane phase. The highest Cα peak at 53 ppm serves as an indicator of the protein backbone dynamics. The four membrane-bound tau samples exhibit C-H order parameters of 0.60 to 0.70 at 500 μs CP, which increase to 0.8 to 1.0 when the CP contact time shortens to 70 μs^49^. Among the four membranes, the cholesterol-containing SUV sample exhibits the largest order parameters, consistent with fibril formation in this membrane. Interestingly, only modest mobility differences are observed between −2 °C and 15 °C, indicating that the membrane phase does not have a significant impact on the dynamics of bound tau.

## Discussion

### Cholesterol-containing high-curvature membranes induce tau amyloid fibrils

The main finding of this study is that high-curvature cholesterol-containing lipid membranes converted soluble tau to β-sheet amyloid fibrils (Fig. 2a), whose rigid core contains the R3 hexapeptide motif ^306^VQIVYK^311^. Cholesterol and high membrane curvature are both necessary for inducing these tau fibrils. When the vesicle sizes increase to LUVs and MLVs, and when cholesterol is removed from the membrane, no fibrils are observed (Fig. 2b–d). The identity of the β-sheet core of the SUV-induced tau fibrils is partially manifested by the 2D NMR spectra. The observation of a Tyr peak with identical chemical shifts as Y310 in heparin-fibrillized tau, and the presence of more than 6 Gly peaks in the 2D ^15^N-^13^C correlation spectra, indicate that the membrane-induced tau fibril core includes the R3 domain and its two flanking PGGG motifs. The ^306^VQIVYK^311^ hexapeptide motif is the essential amyloidogenic element of tau^50^. The methyl-rich sidechains in this segment have known β-sheet propensity and engage in sidechain interactions with neighboring β-sheets^46^. The PGGG segment of R3 is conserved in all brain tau fibrils known so far^7,9–11^, suggesting that this flexible segment is important for controling the three-dimensional fold of the fibril core. We observed strong Lys sidechain signals in the freshly prepared SUV sample, whereas hydrophobic Ile and Val residues have weak signals. This suggests that tau association with the SUV is initiated by electrostatical attraction between the Lys sidechains and the lipid headgroups. Lys residues are distributed throughout the protein (Supplementary Fig. 4b), for example within the hexapeptide motif (K311) and the ^382^AKAK^385^ segment in R’. This electrostatic attraction may be further strengthened by polar interactions of the Lys ammonium group with the cholesterol hydroxyl group.

**Fig. 6:**
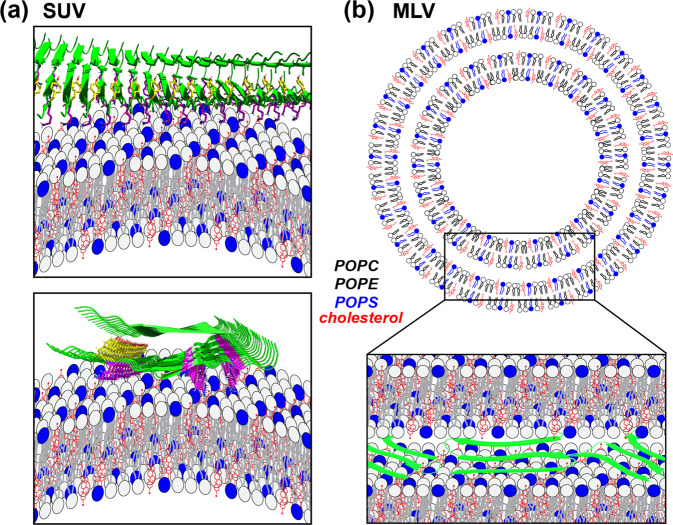
Model of membrane-induced tau β-sheet assembly. **a** Binding tau monomers to preformed cholesterol-containing SUVs promote the formation of amyloid fibrils that contain the hexapeptide motif VQIVYK in the R3 domain. These fibrils are immersed at the membrane-water interface, without inserting into the hydrophobic interior. Key Lys sidechains may face the lipid phosphates to stabilize tau interactions with the lipid membrane. The crucial Y310 residue (yellow) is hypothesized to stack with sidechains from another β-sheet. **b** Tau-lipid co-assembled multilamellar vesicles induce β-strands between adjacent bilayers, without forming ordered amyloid fibrils.

Negative-stain TEM images (Fig. 2a, Supplementary Fig. 2c) of dilute static solutions of tau SUV mixtures show that ~15 nm wide fibrils formed after several weeks. These filaments extend from the SUV surface into the solution for hundreds of nanometers to a micron. Thus, cholesterol-rich SUVs induced tau fibrils both in dilute static solution and in the concentrated environment of the NMR rotor that is subject to spinning. 2D ^1^H-^13^C correlation spectra (Fig. 5) indicate that the immobilized β-sheet domain of SUV-bound tau do not insert into the hydrophobic interior of the lipid bilayer, but is associated with the membrane–water interface. Consistently, TEM images show that the SUV-tau fibrils in the rotor are more confined to the vesicle surface compared to the dilute solution (Fig. 2a), as expected. The most likely fibril orientation that is consistent with these observations is a parallel orientation of both the fibril axis and the β-sheet plane relative to the membrane surface (Fig. 6a). This in-plane orientation would allow the cationic Lys sidechains to intercalate to the membrane-water interface, forming favorable electrostatic interactions with the negatively charged phosphate groups and POPS headgroups. Two β-sheets, separated by 1 nm, could lie on the membrane surface in this way, allowing sidechain steric zippers to form. Based on the observed β-sheet Tyr signal, we hypothesize that Y310 may reside near the membrane plane in the SUV-induced fibrils. An alternative protein orientation is for the fibril axis to lie perpendicular to the membrane surface while the β-strand axis lies parallel to the membrane plane. This model would allow 2–3 β-strands to reside at the membrane-water interface, since two adjacent backbones are separated by 4.8 Å. However, this structural model is less likely because a long fibril would have only a minimal interaction with the membrane vesicle. Moreover, both polar and hydrophobic sidechains of the first two or three rungs of the β-sheet would be similarly immersed at the membrane-water interface, which is energetically unlikely.

In contrast to SUVs, MLVs, LUVs and cholesterol-free SUVs did not induce amyloid fibrils but formed other β-sheet assemblies. Chemical shift assignment indicates that the β-sheet cores of MLV- and LUV-bound tau both include the Ala-containing R’ domain. Both samples may also contain one of the PGGG motifs, since several Gly signals are observed in the 2D NCα spectra and Pro signals are present in the 2D CC spectra. The inclusion of the R’ domain in these low-curvature membranes is reminiscent of microtubule-bound tau^18^. On taxol-stabilized microtubules, the R4 PGGG motif that precedes the R’ is immobilized; but when in contact with dynamically unstable microtubules, this R4 PGGG motif becomes disordered. Thus, the rigidity of the C-terminal half of the R4 domain is sensitive to the nature of the binding partner. Microtubules are cylindrical objects with zero curvature along the tubule axis and 1/r curvature in the transverse plane that contains the 13 tubulin monomers^51^. In contrast, spherical lipid vesicles with a radius *r* have a 1/*r* curvature in all directions. Tau is known to bind microtubules like a ribbon along the tubule axis, suggesting that R’ might not sense curvature^12^. The current finding that R’ binds LUVs and MLVs but not SUVs is consistent with this R’ anchoring to microtubules. Thus, this fifth repeat of tau appears to preferentially interact with low-curvature anionic surfaces. The effect of flat surfaces on nucleation of tau fibrils was also suggested by a recent study that reported the aggregation of full-length tau in the presence of polytetrafluoroethylene beads^52^.

The role of cholesterol in mediating amyloid protein interactions with lipid membranes has long been of interest, although different effects have been observed for different proteins. Cholesterol slowed down membrane-induced amyloid formation of the islet amyloid polypeptide^53^ but accelerated fibril formation by the Aβ peptide^54^. These opposite effects may reflect distinct interactions between cholesterol and the protein of interest. Cholesterol-binding residues in the Aβ peptide and its parent protein, the amyloid precursor protein, have been experimentally studied^55^ and computationally simulated^56^. Through these specific interacting residues, cholesterol might facilitate the clustering of Aβ peptides on the membrane surface to nucleate fibril formation. Future studies will be required to address whether specific cholesterol-recognition motifs exist in tau to nucleate amyloid formation.

What is the cause of the different β-sheet assemblies of tau by SUVs on the one hand and LUVs and MLVs on the other? We hypothesize that these two membrane morphologies present different hydrophobic interactions, which lead to different β-sheet assemblies. Sandwiched by two lipid bilayers in the MLVs (Fig. 6b), the Ala-containing R’ might provide strong hydrophobic interactions with the membrane surface. But on the water-exposed surface of the high-curvature SUVs, tau might recruit the less hydrophobic R3 repeat to nucleate cross-β amyloid formation. The flanking PGGG segments before and after the R3 hexapeptide might further promote this β-sheet core by conforming to the curvature of the SUVs. The fact that LUV-bound tau did not form amyloid fibrils further underscores the importance of high membrane curvature for nucleating fibrils of wild-type tau. We hypothesize that the accumulation of tau in synapses, where high-curvature small synaptic vesicles abound, in early stages of tauopathies might facilitate the nucleation of tau aggregates in the brain^26^.

Despite differences in curvature and cholesterol content, all four membranes converted monomeric random coil tau to β-sheet rich conformations, without detectable amounts of α-helical conformation. The β-rich conformation is consistent with previous findings of various tau constructs bound to negatively charged lipid bilayers such as DMPC/DMPS^57^ and monolayers such as DMPG^58^. We attribute this β-sheet propensity to the two-dimensional nature of the lipid membrane, the condensing effect of the membrane surface, and the highly charged character of tau, which inhibits its insertion into the hydrophobic core of the lipid bilayer. Depending on the specific balance between polar and hydrophobic interactions, and the membrane curvature, different β-sheet assemblies are induced by the membrane. LUV and MLV-bound tau has a high β-sheet content but does not form extended hydrogen-bonded amyloid fibrils, whereas SUV-bound tau has a lower fraction of β-sheet residues but forms well-ordered amyloid fibrils.

### Comparison with other tau-membrane studies and with α-synuclein

Several previous studies of tau-lipid interactions reported the observation of fibrils; however, they did not give residue-specific information about the nature of these filaments nor used lipid mixtures that mimic biological membranes. When full-length 2N4R tau was bound to porcine brain phosphatidylserine (BPS) vesicles, electron micrographs showed curved filaments^59^, but the molecular conformation of these filaments was not known. More recent studies using the same BPS vesicles did not detect filaments but instead observed small homogeneous particles composed of a mixture of protein and lipids^60^. Amino acid type assignment of 2D ^13^C-^13^C correlation NMR spectra identified Val, Ile, and Lys signals, suggesting that the R3 hexapeptide may be included in the rigid core of these protein-lipid particles. These different results from the same BPS vesicles suggest that pure anionic membranes might be deficient in reproducibly inducing tau fibrils, and zwitterionic lipids and cholesterol may be important for stabilizing both hydrophobic and polar sidechains at the membrane-water interface.

In a more substantial departure from bilayer-bound tau, anionic detergents such as SDS were found to give rise to predominantly α-helical chemical shifts of tau^61^. This finding was interpreted as a helix-induced clustering of tau on the micelle surface so that the more amyloidogenic segments might align to form hydrogen-bonded β-sheets^50,62^. These disparate observations obtained from simplified membrane mimetics underscore the importance of using phospholipid bilayers to identify the structural evolution of tau in a cellular context.

Another important amyloid protein whose membrane interaction has been extensively studied is α-synuclein. While tau binds and stabilizes microtubules, α-synuclein is thought to maintain neurotransmitter release by binding to and regulating synaptic vesicles^63^. Aggregation of α-synuclein into amyloid fibrils occurs in Parkinson’s disease (PD)^64^ and other neurodegenerative disorders^65^. Importantly, the Lewy body that is the hallmark of PD contains a mixture of α-synuclein and lipids, indicating that lipids are central to α-synuclein pathology. Solid-state NMR studies showed that α-synuclein co-sediments with SUVs made of a mixture of zwittrionic and anionic lipids, similar to the behavior of tau found here. However, SUV-bound α-synuclein initially adopts an α-helical conformation^66^, in contrast to tau. Time-dependent changes of the SUV-bound α-synuclein conformation have also been studied using solid-state NMR^67,68^. The data show that many residues convert from an α-helical to a β-sheet conformation, and this change occurs in cholesterol-free anionic SUVs. Importantly, α-synuclein shows lipid–protein cross peaks^67,68^ in 2D NMR spectra, in contrast to tau, indicating that α-synuclein interacts intimately with lipid acyl chains after incubation. Recently, cryo-EM structures of lipid-induced α-synuclein fibrils prepared at high P/L ratios were reported. These data show clusters of lipid molecules in a micelle-like state, either bridging protofilaments or covering the surface of the filaments. MD simulations suggest that these lipid molecules may use their acyl chains to stabilize hydrophobic residues and their polar headgroups to stabilize cationic residues^69^. Our 2D ^1^H-^13^C correlation spectra show a complete absence of lipid acyl chain cross peaks with tau, even after extended equilibration (Fig. 5). This indicates that unmodified tau does not have close contact with the hydrophobic portion of lipids, thus tau has a distinct mode of lipid interactions from α-synuclein.

### Implications of membrane-induced tau fibrils on the intercellular spread of tau aggregates

The induction of tau amyloid fibrils by cholesterol-containing small unilamellar vesicles has implications about the mechanism of tau fibril formation in neurons. While tau hyperphosphorylation is known to trigger tau dissociation from microtubules^2,70,71^, the mechanism by which disordered soluble tau monomers nucleate β-strand aggregates is still poorly understood. The current data suggest that high-curvature lipid membranes might provide a mechanism for this nucleation. Immunolabeling data have been reported that show that tau accumulates in presynaptic terminals and impairs its function by binding synaptic vesicles^27^, which have similar sizes to the smallest SUVs used here (~50 nm). Both WT and pathological mutant tau co-sediments with synaptic vesicles, indicating that the membrane-binding properties are independent of disease mutations. Interestingly, N-terminal truncation of tau does not impair synaptic function^27^. Our present data indicate that the N-terminal truncated tau still binds SUVs, consistent with previous solution NMR data that the C-terminal microtubule-binding region of tau is capable of interacting with lipid membranes^61^. Future experiments should investigate whether and how the N-terminal domain changes the kinetics of membrane-induced conformational change of tau from an intrinsically disordered state to a β-sheet fibrillar state, and how the equilibrium structure of membrane-bound tau might differ in the absence and presence of the N-terminal domain.

The present data also place constraints on the mode of translocation of tau species across lipid membranes. Elucidating the molecular mechanisms of intercellular transmission, membrane translocation, and the exact tau species that cross membranes, is crucial for understanding how pathological tau spreads in AD brains^6^. Tau has been reported by immunofluorescence microscopy data to be secreted to the extracellular compartment via both vesicular pathways such as ectosomes^72^ and direct crossing of the plasma membrane in an ATP-independent manner^73,74^. The present data indicate that in the absence of the N-terminal domain, unphosphorylated tau, once assembled in the β-sheet form, does not insert into the hydrophobic interior of 30% negatively charged lipid membranes at neutral pH. Thus, direct crossing of the plasma membrane may require the N-terminal domain, phosphorylation, or the soluble or oligomeric states of tau. Aggregated tau is more likely to adopt the vesicular transport pathways for intercellular communication.

This study examines the impact of membrane curvature and membrane cholesterol on the formation of tau amyloid fibrils and other β-sheet aggregates. Except for SUV-bound tau, which shows narrow linewidths, the other three membrane tau samples—LUV, MLV and cholesterol-free SUV—have broader linewidths compared to full-length 4R and 3R tau fibrils formed in solution^39,41^. We attribute these broad linewidths to the potential distribution of protein states in these samples, the intrinsic dynamic heterogeneity of the protein^39^, and lipid-induced disorder. The LUV and MLV samples may contain oligomeric tau species and even residual monomers, in addition to β-sheet aggregates. In addition, the LUV and MLV-bound tau aggregates may be polymorphic. Because the membrane tau samples studied here were co-sedimented with lipids after a short period of incubation, a coexistence of oligomeric species and larger aggregates of tau is likely, and might reflect the situation of tau interactions with lipid membranes in cells. Future higher-resolution structure characterization of membrane-bound tau may require the selection for a specific tau species and suitable truncation of the protein length, to enhance spectral resolution and spectral sensitivity.

## Methods

### Cloning, expression, and purification of P2R tau

The gene encoding P2R tau contains an N-terminal His_6_ tag, a thrombin cleavage site (10 residues), a TEV cleavage site (6 residues), and residues 198-399 of tau. This gene was cloned into a pET-28a vector and transfected into *E. coli* BL21(DE3) competent cells (New England Biolabs). A starter culture was grown in 50 mL LB medium containing 50 µg/mL kanamycin. After overnight growth at 37 °C with 220 rpm shaking, the 50 mL culture was used to inoculate 1 L of LB medium containing 50 µg/mL kanamycin. Cells were grown at 37 °C and 220 rpm until OD_600_ reached 1.0, then spun down at 1000 g and 4 °C for 20 min. The resulting cell pellet was suspended in 1 L minimal media containing M9 salts, 1 g/L ^15^NH_4_Cl, 2 g/L ^13^C-labeled glucose, 1 mM MgSO_4_, 0.1 mM CaCl_2_, 50 µg/mL kanamycin, vitamin and mineral supplements. Cells were grown in this minimal media at 37 °C under 220 rpm shaking for 2 h, then protein expression was induced with 1 mM IPTG. Another 1 g/L of ^13^C-labeled glucose was added at this point to the medium to reach 3 g/L ^13^C-labeled glucose. Expression proceeded for 5 h at 37 °C under 250 rpm shaking.

The His_6_-thrombin-TEV-P2R tau fusion protein was purified using Ni^2+^ affinity column chromatography. The His_6_-thrombin-TEV tag was cleaved using TEV protease, and the native P2R tau was purified by reverse-phase HPLC. In more detail, cells were spun down at 6000 g and 4 °C for 15 min. The pellet was resuspended in 40 mL of a lysis buffer containing 1× PBS (pH 7.4), 2 mM DTT, 0.05 mM PMSF, and 1 tablet of cOmplete™ protease inhibitor cocktail (Roche). The cells were lysed by sonication on ice (5 s on, 5 s off for 10 min, 550 Sonic Dismembrator, Fisher Scientific), then the lysate was boiled for 10 min and spun at 20,000 g for 60 min to remove cell debris and aggregates of heat-sensitive proteins. The supernatant containing the fusion protein was loaded onto 5 ml of Profinity IMAC resin that had been charged with Ni^2+^ (Bio-Rad, California, USA) and washed with 20 column volumes of a binding buffer (1× PBS, 2 mM DTT, 20 mM imidazole, pH 8). The protein was then eluted using 5 column volumes of the elution buffer (1× PBS, 2 mM DTT, 400 mM imidazole, pH 8). The eluted fraction was loaded onto a desalting column (Econo-Pac 10 DG, Bio-Rad) to exchange buffer for the TEV cleavage buffer (50 mM Tris, 150 mM NaCl, 2 mM DTT, 0.05 mM EDTA, 0.05 mM PMSF, pH 8). The solution was diluted to ~0.5 mg/mL of the fusion protein to prevent aggregation during cleavage. TEV protease was added at a 3: 100 (w: w) ratio with respect to the fusion protein, and the cleavage reaction proceeded for 16 h at 4 °C with gentle rocking. After cleavage, 3 M guanidinium chloride was added to the solution and loaded onto 5 ml of Ni^2+-^charged resin that had been pre-washed with the binding buffer. The P2R tau—containing flowthrough was collected and purified on a reverse-phase HPLC using a preparative C3 column (250 mm length, 21.2 mm I.D., 7 µm particle size, 300 Å pore size, Higgins analytical) and an acetonitrile gradient of 5–95% in 35 min. Immediately before injecting the crude solution into the HPLC, we added a few drops of TFA to the solution to reach pH ~4 to protect the integrity of the column’s silica. The eluted P2R tau fractions were >95% pure as assessed by SDS-PAGE with Coomassie blue staining. The fractions were pooled and lyophilized to give the P2R tau powder. The yield of the expression and purification was ~15 mg protein per liter of M9 culture.

### Preparation of tau-bound membrane samples

Two membrane compositions are used in this work. The cholesterol (chol)-containing membrane contains 1-palmitoyl-2-oleoyl-sn-glycero-3-phosphocholine (POPC), 1-palmitoyl-2-oleoyl-sn-glycero-3-phosphoethanolamine (POPE), 1-palmitoyl-2-oleoyl-sn-glycero-3-phospho-L-serine (POPS) and cholesterol at a molar ratio of 30: 25: 25: 20. The cholesterol-free lipid membrane contains POPC, POPE, POPS at a molar ratio of 38: 31: 31. All lipids (Avanti Polar Lipids) were codissolved in a 1:1 chloroform: methanol solution, then dried with a stream of nitrogen gas and lyophilized overnight to a homogenous film. The cholesterol-containing membranes were mixed with P2R tau at a protein/lipid molar ratio (P/L) of 1:80, which corresponds to 4 mg protein with 12 mg lipids. The cholesterol-free membranes were mixed with the protein at a P/L ratio of 1: 60, which corresponds to 4 mg of protein with 8 mg lipids.

These two membrane compositions were used to prepare four types of proteoliposomes: SUVs, MLVs, LUVs, and cholesterol-free SUVs. We used extended sonication to prepare SUVs, freeze-thawing and coincubation with the protein to prepare MLVs, and extrusion to prepare LUVs. In detail, to prepare the SUV samples, we suspended the lipid mixture in a pH 7.5 buffer (20 mM Tris, 2 mM EDTA, 15 mM NaCl, and 1 mM TCEP or DTT) at 2 mg/ml in a 20 ml glass scintillation vial, vortexed and sonicated it for three cycles of 30 s/30 s, and left it at room temperature for one hour. During this time the tubes were gently flipped a few times every 15 min to avoid sedimentation of the mixture. The suspension was next subjected to five freeze-thawing cycles between liquid nitrogen and a 35–40 °C water bath, followed by another five cycles of freeze-thawing alternating with sonication in a bath sonicator at 24 °C for 45 s each. At the end of this procedure, the vesicle solution appeared translucent. A 2 mg/ml solution of P2R tau monomers was added to about 6 ml of SUVs at a concentration of ~2 mg/ml, to reach a final P/L molar ratio of 1: 80. The solution was incubated for 18 h at 37 °C under 300 rpm shaking (2.5 cm diameter orbit, Excella E25, New Brunswick) before being centrifuged to collect a membrane pellet.

To produce the MLV sample, we suspended the lipid mixture in the pH 7.5 buffer to 2 mg/mL, vortexed and sonicated it for three cycles of 30 s/30 s, then added the 2 mg/ml P2R tau monomer solution to the vesicles to reach a P/L of 1:80. The mixture was left at room temperature for one hour with occasional agitation. The suspension was next subjected to ten freeze-thaw cycles between liquid nitrogen and a 32 °C water bath. This procedure produces multilamellar vesicles where the protein is integrated in between lipid bilayers.

To produce the LUV sample, we suspended the lipid mixture in the pH 7.5 buffer to 4 mg/mL, vortexed and sonicated it for three cycles of 30 s/30 s to obtain a homogeneous solution, then left the solution at ambient temperature for one hour with occasional agitation. The suspension was subjected to five freeze-thaw cycles between liquid nitrogen and 32 °C, after which it was extruded three times through a 400 nm filter and nine times through a 200-nm filter (Avanti® Mini-Extruder). A 2 mg/ml solution of tau monomers was then added to these LUVs at an estimated concentration of 2 mg/ml to reach a P/L of 1: 70. The solution was incubated for 18 h at 37 °C under 250 rpm shaking.

All proteoliposome solutions were centrifuged at 112,000 × *g* using a Beckman TLA55 fixed-angle rotor, then the membrane pellet was slowly dried in a desiccator until the pellet reached ~50% (w/w) water by mass relative to the total mass. The hydrated membranes were packed into 3.2 mm Revolution NMR pencil rotors, 3.2 mm Bruker or 4 mm Bruker rotors for NMR experiments.

### Transmission electron microscopy

Negative-stain transmission electron microscopy (TEM) was used to assess the morphology of the membrane-bound tau. Samples were adsorbed onto freshly glow-discharged, 200-mesh formvar/carbon-coated copper grids (Ted Pella), washed with water, and stained with 0.7% (wt/vol) uranyl formate for 10 s. TEM images were taken on an FEI Tecnai T12 electron microscope. To measure TEM images of samples that have been spun for extended periods of time in MAS rotors, we removed 1 μl of sample from the rotor using a 200 μl pipet tip, resuspended the material in 20 μl of buffer, pipetted it extensively, and vortexed it for 30 s. The solutions were then applied to TEM grids. For freshly prepared samples, unconcentrated solutions of tau-bound vesicles were directly applied to grids. To obtain fibril widths from TEM data, we sampled all particles in multiple images. This is sufficient for the qualitative conclusions made in this study. Similarly, to estimate membrane vesicle diameters from TEM images, we sampled all vesicles in multiple images. This is sufficient for the qualitative conclusions made in this study.

### Solid-State NMR experiments and spectral analysis

Solid-state NMR spectra were measured on three spectrometers: a Bruker Avance 800 MHz (18.8 T) equipped with a BlackFox 3.2 mm HCN probe; an AVANCE III HD 600 MHz spectrometer equipped with a 3.2 mm MAS probe; and an AVANCE III HD 400 MHz spectrometer equipped with a 4 mm MAS probe. Reported sample temperatures were estimated using the water ^1^H chemical shift on the DSS scale^75^. ^13^C chemical shifts were referenced externally to the adamantane CH_2_ chemical shift at 38.48 ppm on the tetramethylsilane scale. ^15^N chemical shifts were referenced to the ^15^N peak of N-acetylvaline at 122.00 ppm on the liquid ammonia scale. More detailed experimental parameters are given in Supplementary Table 1.

Static ^31^P spectra of lipid membranes were measured on the 400 MHz spectrometer using a 4 mm HXY probe operating in the ^1^H/^31^P double-resonance mode. For the uniaxial averaged static ^31^P powder pattern, the chemical shift span is defined as the difference between the downfield edge σ_//_ to the upfield edge ${\sigma }_{\perp }$^76–79^.

2D ^13^C–^13^C dipolar correlation spectra were measured using Combined-Driven (CORD) spin diffusion^80^ for ^13^C mixing. 2D ^15^N-^13^C dipolar correlation spectra were measured using SPECIFIC cross polarization (CP)^81^ for ^15^N-^13^C polarization transfer. A Black Fox low-E probe^82^ was used for these experiments because the crossed-coil dual resonator has higher radiofrequency (rf) field homogeneity than solenoid coils, giving four-fold higher ^15^N-^13^C SPECIFIC-CP efficiencies compared to Bruker HCN probes. The 2D NC spectra were measured under 10.5 kHz or 14 kHz MAS. Typical rf field strengths were 70–90 kHz for ^1^H, 30–50 kHz for ^13^C, and 25–40 kHz for ^15^N.

1D and 2D INEPT ^1^H-^13^C spectra were measured to detect the signals of dynamic residues. ^1^H-^13^C CP spectra were measured using CP contact times of 500 μs or 1 ms to preferentially detect rigid and semi-rigid residues. For all 2D spectra, multiple blocks were recorded and added in the time domain before Fourier transformation. Spectra were processed using GM apodization in the Topspin software, and chemical shift assignment was conducted using CCPNMR^83^.

2D ^1^H-^13^C heteronuclear correlation (HETCOR) experiments were carried out at 290 K under 10.5 kHz MAS^48,84,85^. After ^1^H excitation, a ^1^H T_2_ filter with a total echo period of 2 × 2.18 ms was applied in which the ^1^H 180° pulse was applied simultaneous to a ^13^C 180° pulse. This ^1^H T_2_ period selects the water and lipid ^1^H magnetization for later transfer to the protein. At the same time, the ^13^C 180° pulses reintroduces the ^13^C-^1^H scalar coupling to suppress the ^1^H magnetization of the ^13^C-labeled proteins while retaining the ^1^H magnetization of the natural abundance lipids^84,85^. The selected water and lipid ^1^H magnetization was next transferred to the protein via chemical exchange and spin diffusion during a z filter. The protein ^1^H magnetization is finally cross-polarized to ^13^C for detection. At a ^1^H mixing time of 0, no ^13^C signals were detected, indicating that the protein ^1^H magnetization was initially removed by the T_2_ filter. At a mixing time of 100 ms, the ^13^C spectra show the same intensity pattern as the 1D CP spectra but with only half the intensity, indicating that the water and protein ^1^H magnetization is equilibrated by 100 ms.

2D ^1^H-^13^C dipolar-chemical-shift (DIPSHIFT) correlation experiments were conducted under 10.5 kHz MAS at a sample temperature of −2 °C and 15 °C to assess the protein mobility^86^. The ^1^H-^13^C CP contact times were 70 μs and 500 μs. The ^13^C magnetization was dephased by ^1^H-^13^C dipolar coupling during the doubled DIPSHIFT period^87^. ^1^H-^1^H homonuclear decoupling was achieved using the FSLG sequence, whose theoretical scaling factor is 0.577^88^. The measured ^13^C intensities at various dephasing times was suitably integrated and normalized to the intensity of the first time point. Theoretical DIPSHIFT curves were simulated for varying dipolar order parameters, S_CH_, using a custom-written python code. Simulations used a rigid-limit FSLG-scaled C-H dipolar coupling of 26.34 kHz.

### Statistics and reproducibility

To assess membrane vesicle diameters from TEM images, all vesicles in multiple images were sampled. This is sufficient for the qualitative conclusions in this study. To assess fibril widths from TEM data, all fibrils in multiple images were sampled. This is sufficient for the qualitative conclusions in this study. Biochemical characterization, including sedimentation assay and TEM, was performed twice prior to preparing NMR samples. Each NMR sample was made once and the NMR spectra were measured over the course of several months on the same set of samples. Each NMR sample contained more than 10^16^ molecules, thus the NMR spectra report the average over all molecules in the sample. A large number of scans were averaged for each spectrum to obtain sufficient signal-to-noise ratios. Sample size was chosen by maximizing the amount of material that could be fit into the sample rotor.

### Reporting summary

Further information on research design is available in the Nature Portfolio Reporting Summary linked to this article.

## Supplementary information

Peer Review File

Supplementary Information

Reporting Summary

## Data Availability

All data needed to evaluate the conclusions are provided in the paper and the Supplementary Materials.

## Code availability

This work does not contain specialized source codes.
