# Supplementary material for: Membrane-induced tau amyloid fibrils
Source: Commun Biol. 2023 Apr 28;6:467. doi: 10.1038/s42003-023-04847-6 (PMC10147698; doi:10.1038/s42003-023-04847-6)
Supplement: Supplementary file 2 — Supplementary Information [file 42003_2023_4847_MOESM2_ESM.pdf]

Supplementary Information

Membrane-Induced Tau Amyloid Fibrils

Nadia El Mammeri, Olivia Gampp, Pu Duan, and Mei Hong*

Department of Chemistry, Massachusetts Institute of Technology, 170 Albany Street, Cambridge, MA 02139

* Corresponding author:
Professor Mei Hong. Email: meihong@mit.edu; Tel: 616-253-5521

This PDF file includes:
Supplementary Figures 1 – 7
Supplementary Tables 1 – 4

Supplementary Table 1. Solid-state NMR experimental parameters of membrane-bound tau. Spectra were measured on 800 MHz (18.8 T), 600 MHz (14.1 T), and 400 MHz (9.4 T) spectrometers. Reported temperatures are estimated sample temperatures based on the water ^{1}H chemical shift.

**Experiment**	**NMR Parameters**	**Samples**	**Expt. Time**
2D CC, 50-100 ms CORD mixing	T_{sample} = 271 K; ν_{MAS} = 10.5 kHz, τ_{rd} = 1.5 s, $t_{1,max}$ = 8.7 ms; $t_{1,inc}$ = 24.8 μs; τ_{dwell} = 6 μs; τ_{acq} = 15.4 ms; τ_{HC} = 500 μs; τ_{CORD} = 50-100 ms; ν_{1Hacq} = 71 kHz TPPM	SUV POPX chol : P2R<br>MLV POPX chol : P2R<br>SUV POPX : P2R<br>LUV POPX chol : P2R	54 hrs<br>36 hrs<br>72 hrs<br>36 hrs
2D NCA, specific CP	T_{sample} = 271 K; ν_{MAS} = 10.5 kHz, τ_{rd} = 1.7 s, $t_{1,max}$ = 9.5 ms; $t_{1,inc}$ = 142.8 μs; τ_{dwell} = 6 μs; τ_{acq} = 15.4 ms; τ_{HN} = 1 ms; τ_{NC} = 5 ms; $\nu_{15NspecificCP}$ = 26.3 kHz ramp 90-100%; $\nu_{13CspecificCP}$ = 15.8 kHz; $\nu_{1HspecificCP}$ = 71 kHz CW; ν_{1Hacq} = 71 kHz TPPM	SUV POPX chol : P2R<br>MLV POPX chol : P2R<br>SUV POPX : P2R<br>LUV POPX chol : P2R	2 days<br>1 day<br>1 day<br>2 days
2D N(CA)CX specific CP, 50 ms CORD mixing	T_{sample} = 271 K; ν_{MAS} = 14 or 10.5 kHz, , τ_{rd} = 1.7 s, $t_{1,max}$ = 6.4 ms; $t_{1,inc}$ = 142.8 μs; τ_{dwell} = 6 μs; τ_{acq} = 15.4 ms; τ_{HN} = 1 ms; τ_{NC} = 5 ms; τ_{CC} = 50 ms; $\nu_{15NspecificCP}$ = 25.2 kHz 90-100% ramp; $\nu_{13CspecificCP}$ = 39.2 kHz; $\nu_{1HspecificCP}$ = 71 kHz CW; ν_{1Hacq} = 83 kHz TPPM; $\nu_{1HBSHCP}$ = 90 kHz CW	SUV POPX chol : P2R<br>MLV POPX chol : P2R	90 hrs<br>45 hrs
2D N(CO)CX specific CP, 50 ms CORD mixing	T_{sample} = 271 K; ν_{MAS} = 14 kHz; τ_{rd} = 1.7 s, $t_{1,max}$ = 6.4 ms; $t_{1,inc}$ = 142.8 μs; τ_{dwell} = 6 μs; τ_{acq} = 15.4 ms; τ_{HN} = 1 ms; τ_{NC} = 4.5 ms; τ_{CC} = 50 ms; $\nu_{15NspecificCP}$ = 25.2 kHz 90-100% ramp; $\nu_{13CspecificCP}$ = 39.2 kHz; $\nu_{1HspecificCP}$ = 71 kHz CW; ν_{1Hacq} = 83 kHz TPPM	SUV POPX chol : P2R<br>MLV POPX chol : P2R	90 hrs<br>45 hrs
2D ^{1}H-^{13}C INEPT	T_{sample} = 271 K; ν_{MAS} = 10.5 kHz,192, τ_{rd} = 2 s, $t_{1,max}$ = 15.9 ms; $t_{1,inc}$ = 104.2, 124.8 μs; τ_{dwell} = 7.2, 6 μs; τ_{acq} = 24.6 ms; ν_{1Hacq} = 71 kHz TPPM	SUV POPX chol : P2R<br>MLV POPX chol : P2R<br>LUV POPX chol : P2R	25 hrs<br>14 hrs<br>17 hrs
T_2-filtered J-edited 2D ^{1}H-^{13}C HETCOR	T_{sample} = 271 K; ν_{MAS} = 10.5 kHz, τ_{rd} = 2 s, $t_{1,max}$ = 6.4 ms; $t_{1,inc}$ = 166.6 μs; τ_{dwell} = 6 μs; τ_{acq} = 15.4 ms; τ_{SD}=100ms; τ_{filter}=2.18 x 2 ms; ν_{1Hacq} = 71 kHz TPPM	SUV POPX chol : P2R<br>MLV POPX chol : P2R<br>LUV POPX chol : P2R	18 hrs<br>18 hrs<br>17 hrs
2D CH DIPSHIFT	T_{sample} = 271 or 288 K; ν_{MAS} = 10.5 kHz, ns = ~3072, τ_{rd} = 1.7 s, $t_{1,max}$ = 95.8 μs; $t_{1,inc}$ = 8.24 μs; τ_{dwell} = 6 μs; τ_{acq} = 15.4 ms; ν_{1Hacq} = 83 kHz TPPM	SUV POPX chol : P2R<br>MLV POPX chol : P2R<br>SUV POPX : P2R<br>LUV POPX chol : P2R	16 hrs<br>16 hrs<br>16 hrs<br>16 hrs
1D Tyr ^{13}C CP and Arg ^{15}N CP	T_{sample} = 271 K; ν_{MAS} = 16 kHz, τ_{rd} = 1.7 s, τ_{dwel} = 6 μs; τ_{acq} = 15.3 ms; τ_{HC} = 500 μs; τ_{HN} = 1000 μs: ν_{1Hacq} = 71 kHz TPPM	SUV POPX chol : P2R<br>MLV POPX chol : P2R	10 hr<br>10 hr

Symbols: T_{sample} = sample temperature; ν_{MAS} = MAS frequency; ns = number of scans (transients) per free induction decay (FID); τ_{rd} = recycle delay between scans; $t_{1,max}$ = maximum t_1 (indirect dimension 1) evolution time; $t_{1,inc}$ = increment for t_1 (indirect dimension 1) evolution time; τ_{dwell} = dwell time during direct FID acquisition; τ_{acq} = maximum acquisition time during direct FID detection; τ_{XY} = cross polarization (CP, ^{SPECIFIC}CP) contact time during CP from channel X to channel Y; $\nu_{nuc\text{-}CP,XY}$ = radiofrequency field strength for CP spin lock on *nuc* (*nuc* = ^{1}H, ^{13}C, ^{15}N) during CP from X to Y; $\nu_{1H,\,acq}$ = dipolar decoupling field strength during FID acquisition and indirect dimension evolution; $\nu_{nuc\text{-}DCP}$ = rf spin lock field strength on *nuc* during heteronuclear CP; $\nu_{1H\text{-}DD,DCP}$ = ^{1}H dipolar decoupling field strength during heteronuclear CP.

Supplementary Table 2. ^{13}C and ^{15}N chemical shifts (ppm) of immobilized residues in P2R tau bound to cholesterol-containing SUVs. For residues whose sequential assignments have been obtained, the sequentially assigned motifs are shown in brackets. Ambiguous assignments are italicized.

**Residues**	**N**	**C'**	**Cα**	**Cβ**	**Cγ/γ1**	**Cγ2**	**Cδ/δ1**	**Cδ2**	**Cε/ ε1**
Gly1 (GG)	106.7		42.1						
Gly2 (GG)	108.3	172.0	43.0						
Gly3 (GG)	104.2		42.4						
Gly (GS-*V/L*)			45.3						
Ser (GS-*V/L*)	121.6		55.9	64.0					
Val-Leu (GS-*V/L*)	130.1								
Val (VY)	126.9		59.9	31.1	19.2				
Tyr (VY)	132.0		55.3	39.0					
Ser1	117.2	172.0	53.5	63.3					
Ser2	125.0		56.3	61.8					
Ser3			52.6	62.0					
Ser4			53.4	65.0					
Thr1			58.6	68.5					
Ile1	127.2		57.8	38.7	25.6	15.2	11.8		
Val1	118.7		58.0			19.2			
Val2	126.0		58.9	33.3					
Val3	130.4		59.7						
Asn/Asp1	127.3		50.1	40.6					
Asn/Asp/Leu2			52.2	43.4					
Asn/Asp/Leu3			52.8	41.9					
Gly1	107.2	171.8	41.0						
Gly2	103.1		42.5						
Lys1	119.6		53.0		23.4		28.0		38.9
Pro		174.2	61.3	30.3	25.4		49.3		

Supplementary Table 3. ^{13}C and ^{15}N chemical shifts (ppm) of immobilized residues in P2R tau bound to cholesterol-containing MLVs. For residues whose sequential assignments have been obtained, the sequentially assigned motifs are shown in brackets.

Residues	Cα	Cβ	C'	N	Cγ/γ1	Cγ2	Cδ/δ1	Cδ2	Cε/ ε1
Lys (AK)	53.2	34.5	172.8		23.0		27.7		39.8
Ala (AK)	49.3	21.8		126.2					
Ala2 (AK)	49.6	20.9							
Ser1	56.2	61.5		118.4					
Ser2	53.4	58.4		115.0					
Ser3	54.4	63.8		113.5					
Ser4	57.4	63.0		118.8					
Ser5	55.0	65.6		107.5					
Thr1	59.6	67.5				19.4			
Thr2	58.7	68.9				19.8			
Ile1	57.6	38.9		126.8	25.3	15.3	11.5		
Ile2	58.8	36.2				15.3	10.8		
Val1	58.8	30.5		120.6		19.1			
Val2	58.9	32.7		125.2		19.4			
Val3	60.5	30.1							
Val4	58.2	29.8		117.2		19.6			
Asn/Asp1	50.6	38.8	177.6	126.9					
Asn/Asp2	51.9	43.7							
Asn/Asp3	50.4	40.5							
Leu1	52.1	39.7		129.2	25.7		24.0		
Lys/Arg1	54.1	30.7			22.8				
Ala1	52.2	19.5							
Ala2	49.6	15.9							
Ala3	50.0	17.0							
Ala4	51.1	16.6		122.0					
Pro	60.3	30.0			25.3		48.4		

Supplementary Table 4. Description of the samples shown in Fig. 2, where each row depicts a different sample preparation procedure and each column describes the state of the sample. TEM images of the first and last column in Fig. 2 represent the same $^{13}C/^{15}N$-labelled samples while the 'Static solution, equilibrated' samples were independently prepared. These samples are annotated with an asterisk (*) below.

**Sample ID**			**Maturation procedure**
1	SUV + tau	Static solution, initial	Sample was measured by TEM immediately after preparation, then packed into MAS rotors and measured for ~15 days at 400 MHz at sample temperatures of 264–294 K.
2*	SUV + tau	Static solution, equilibrated	Sample was prepared using unlabeled P2R tau monomers and kept at 37°C for 12 weeks without shaking before measuring TEM data.
3	SUV + tau	After MAS, equilibrated	Sample was kept in the NMR rotor at -20°C, then NMR spectra were measured after 4 weeks and the TEM images were collected after 12 weeks.
4	MLV + tau	Static solution, initial	Sample was measured by TEM immediately after preparation, then packed into an NMR rotor and measured for ~10 days at 800 MHz at sample temperatures of 264–294 K.
5*	MLV + tau	Static solution, equilibrated	Sample was prepared using unlabeled P2R tau monomers and kept at 37°C for 2 weeks without shaking before collecting TEM data.
6	MLV + tau	After MAS, equilibrated	Sample was kept in the NMR rotor at -20°C, then NMR spectra were measured after ~4 weeks and TEM images were measured after ~12 weeks.
7	Chol-free SUV + tau	Static solution, initial	Sample was measured by TEM immediately after preparation, then packed into an NMR rotor and measured for ~10 days at 400 MHz at sample temperatures of 264–294 K.
8*	Chol-free SUV + tau	Static solution, equilibrated	Sample was prepared using unlabeled P2R tau monomers and kept at 37°C for ~12 weeks without shaking before measuring TEM data.
9	Chol-free SUV + tau	After MAS, equilibrated	Sample was kept in an NMR rotor at -20°C for several weeks before measuring the NMR spectra at about 10 weeks and the TEM data at ~12 weeks.
10	LUV + tau	Static solution, initial	Sample was measured by TEM immediately after preparation, then packed into MAS rotors and measured for ~10 days at 400 MHz at sample temperatures of 264–294 K.
11*	LUV + tau	Static solution, equilibrated	Sample was prepared using labeled P2R tau monomers and kept at 37°C for 5 weeks without shaking before measuring TEM data.

12	LUV + tau	After MAS, equilibrated	Sample was kept in the NMR rotor at 24°C, then NMR spectra were measured after 5 weeks and TEM images were collected after 6 weeks.

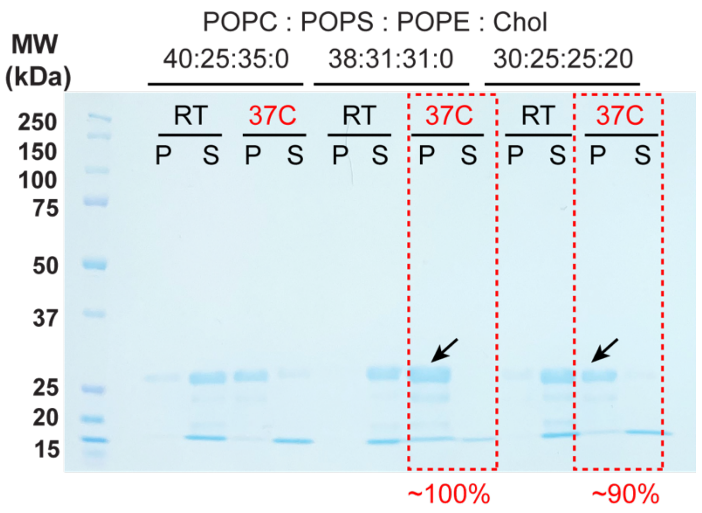

Supplementary Figure 1. **SDS-PAGE gel characterizing the P2R tau purity and binding to lipid membranes.** The protein was added to preformed SUVs with different lipid compositions and at different temperatures. Higher temperature (37˚C) increased the percentage of protein in the pellet (P) than the supernatant (S). Thus, P2R tau co-sediments with lipid vesicles more efficiently at higher temperature with shaking. Based on the gel band intensities, the binding is nearly quantitative at 37˚C, under 300 rpm shaking, in SUVs both with and without cholesterol.

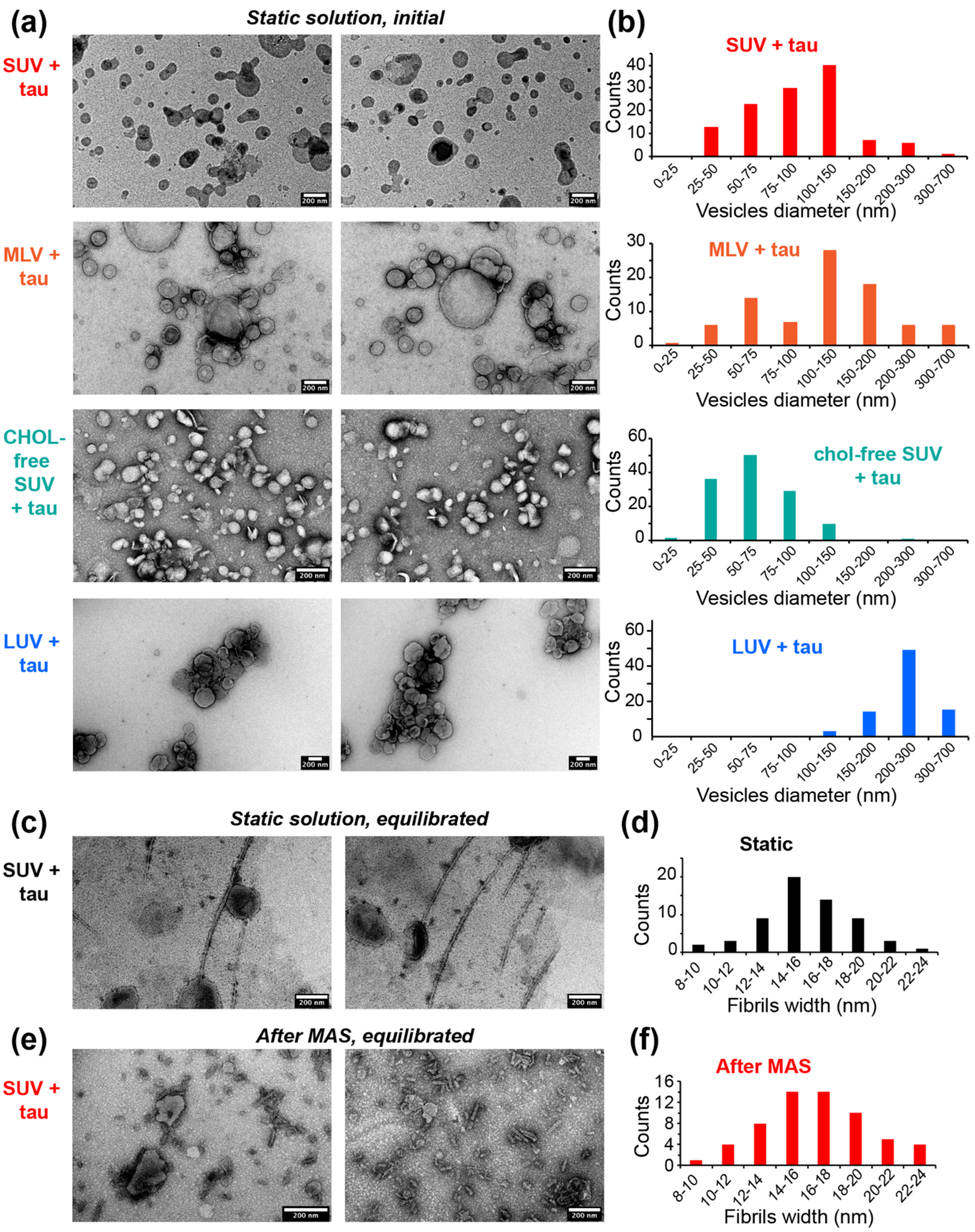

Supplementary Figure 2. Negative-stain TEM images allow quantification of the size of tau-bound membrane vesicles and fibril morphology. (a) TEM images of freshly prepared membrane-tau samples in solution, without shaking or spinning. **(b)** Vesicle size distributions for the SUV, MLV, cholesterol-free SUV, and LUV samples obtained from the images in (a). **(c)** TEM images of equilibrated SUV-bound tau from a static solution. Long fibrils are observed. (**d**) Fibril width distributions obtained from the images in (c). The SUV-induced tau fibrils are 14-18 nm

wide. (**e**) TEM images of equilibrated SUV-bound tau sample after being spun for many weeks. Short fibrils are seen coating on the surface of the vesicles. (**f**) Fibril width distribution of the spun SUV-tau sample. The distribution is similar to that of the static solution sample.

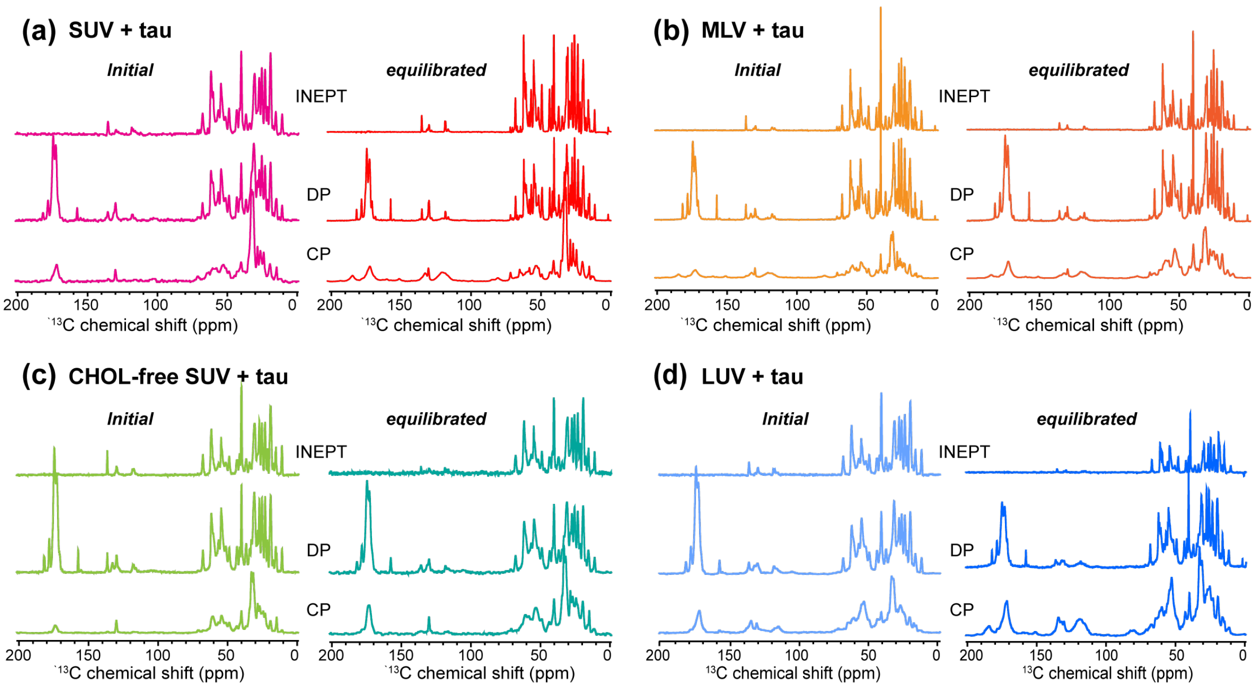

Supplementary Figure 3. **^{13}C MAS spectra of membrane-bound P2R tau at the initial stage and after equilibration**. CP, DP and INEPT spectra were measured for each sample to preferentially detect rigid residues, all residues, and highly mobile residues, respectively. (**a**) SUV-bound P2R tau spectra. The fresh spectra and equilibrated spectra were measured on a 400 MHz and 800 MHz spectrometer, respectively. (**b**) MLV-bound P2R tau spectra. (**c**) Spectra of P2R tau bound to cholesterol-free SUVs, measured on a 400 MHz NMR. (**d**) LUV-bound P2R tau spectra. The initial and equilibrated spectra were measured on the 400 and 800 MHz spectrometers, respectively. The CP intensities increased over time for the MLV, chol-free SUV, and LUV samples. Interestingly, the SUV-bound tau did not show a CP intensity increase, but the residues that give CP intensities in this sample are more rigid and ordered than the other samples, as shown by various 2D spectra. All spectra were measured at a sample temperature of 270 K.

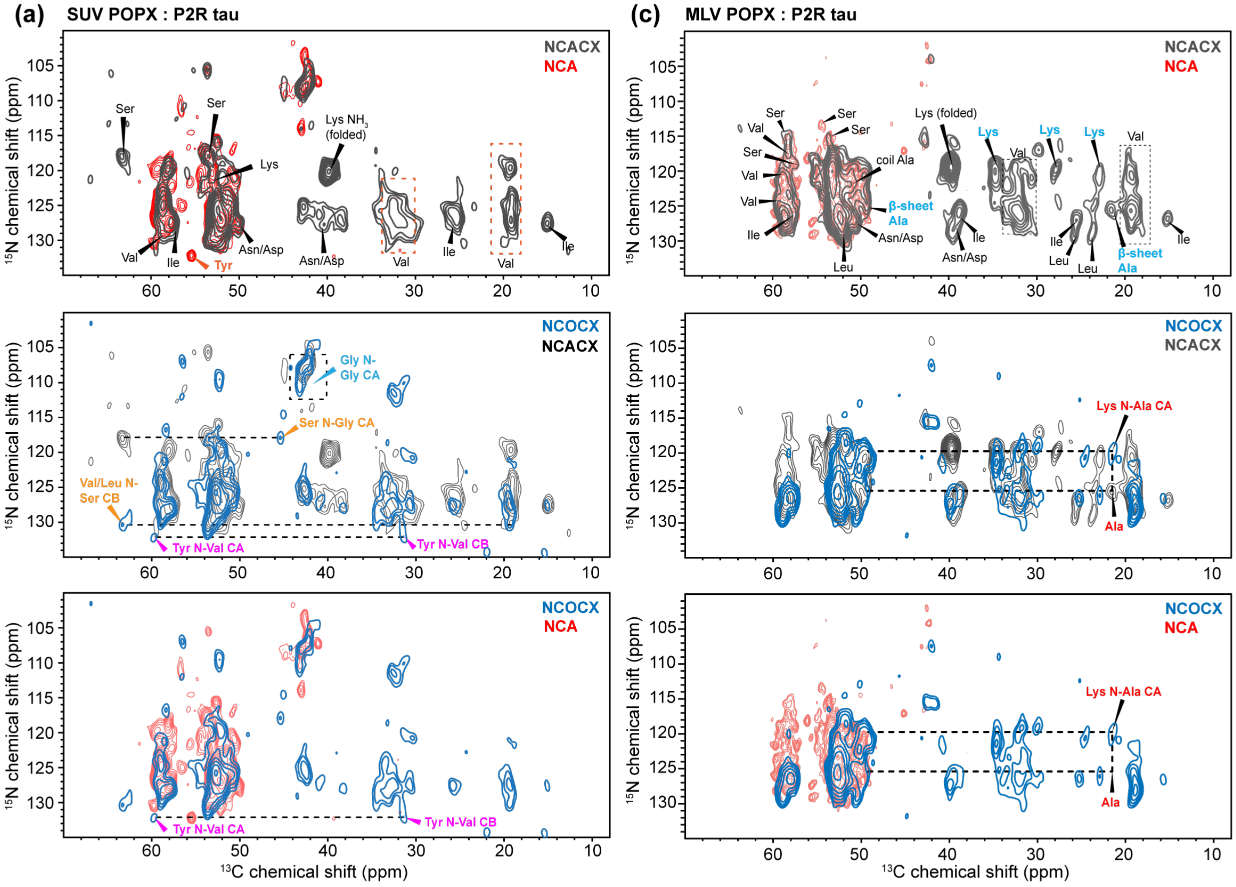

(b)

```
P2 198 SSPGSPGTPG SRSRTPSLPT PPTREPKKVA VVRTPPKSPS SAKSRL 243
R1 244 QTAPVPMPDL KNVKSKIGST ENLKHQPGGG K 274
R2 275 VQIINKKLDL SNVQSKCGSK DNIKHVPGGG S 305
R3 306 VQIVYKPVDL SKVTSKCGSL GNIHHKPGGG Q 336
R4 337 VEVKSEKLDF KDRVQSKIGS LDNITHVPGG GN 368
R' 369 KKIETHKLTF RENAKAKTDH GAEIVYKSPV V 399
```

(d)

```
P2 198 SSPGSPGTPG SRSRTPSLPT PPTREPKKVA VVRTPPKSPS SAKSRL 243
R1 244 QTAPVPMPDL KNVKSKIGST ENLKHQPGGG K 274
R2 275 VQIINKKLDL SNVQSKCGSK DNIKHVPGGG S 305
R3 306 VQIVYKPVDL SKVTSKCGSL GNIHHKPGGG Q 336
R4 337 VEVKSEKLDF KDRVQSKIGS LDNITHVPGG GN 368
R' 369 KKIETHKLTF RENAKAKTDH GAEIVYKSPV V 399
```

Supplementary Figure 4. **Amino acid type assignment and partial sequential assignment of membrane-bound P2R tau**. 2D N(CA)CX, N(CO)CX and NCα spectra are jointly analyzed for this resonance assignment. (**a**) Spectra of equilibrated SUV-bound P2R tau. Top: overlay of the 2D N(CA)CX spectrum (black) with the NCα (red) spectrum; Middle: overlay of the 2D N(CO)CX spectrum (blue) with the N(CA)CX spectrum. Bottom: overlay of the N(CO)CX spectrum with the NCα spectrum. From an N-terminal residue's ^{13}C to its C-terminal residues' ^{15}N, sequential connectivities are found for Gly-Gly, Gly-Ser, Ser-Leu/Val, and Val-Tyr pairs. These residue pairs are assigned in bold. (**b**) Amino acid sequence of P2R tau, with Gly-Gly, Gly-Ser, Ser-Leu/Val, and Val-Tyr pairs highlighted. (**c**) Spectra of equilibrated MLV-bound P2R tau. The spectra are overlaid in the same way as on the left. A sequential Ala-Lys cross peak is found. Three Ala-Lys pairs exist in the protein, one pair in the P2 domain and two pairs in R'. However, P2 is highly mobile based on the INEPT spectra, thus the observed dipolar cross peaks can be assigned to the AKAK motifs in the R' domain. (**d**) Amino acid sequence of P2R tau, indicating the position of the AKAK motif in R'.

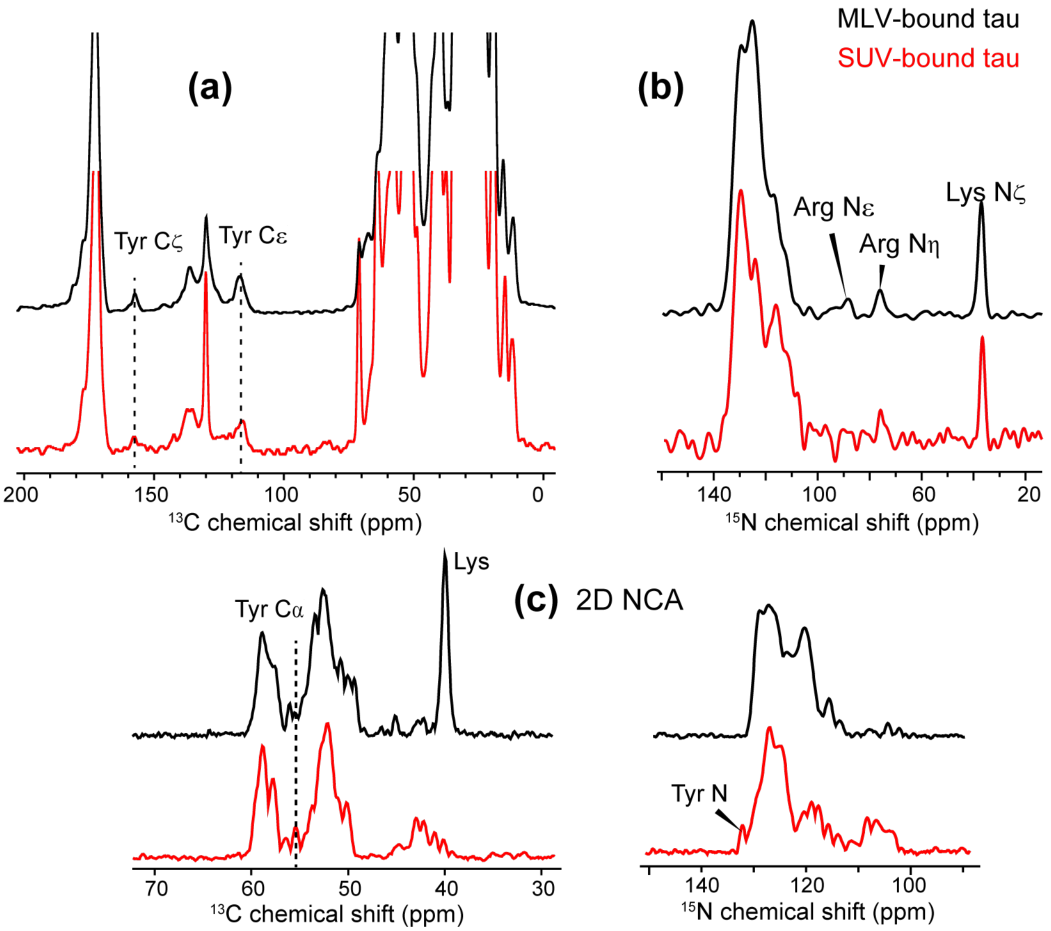

Supplementary Figure 5. Tyr ^{15}N and ^{13}C chemical shifts in 1D and 2D spectra of SUV-bound tau (red) and MLV-bound tau (black). (**a**) 1D ^{13}C CP spectra of equilibrated SUV and MLV samples. The unique Tyr aromatic Cζ and Cε peaks are indicated. (**b**) 1D ^{15}N CP spectra. Note the high intensities of the Lys NH_3 peak compared to the Arg Nε and Nη peaks. (**c**) Positive projections of the 2D NCα spectra of the SUV and MLV samples along the ^{13}C and ^{15}N dimensions. The rigid Tyr ^{15}N and Cα chemical shifts in the SUV spectrum are indicated. The backbone ^{15}N signal of Tyr is not resolved in the 1D ^{15}N spectrum shown in (b).

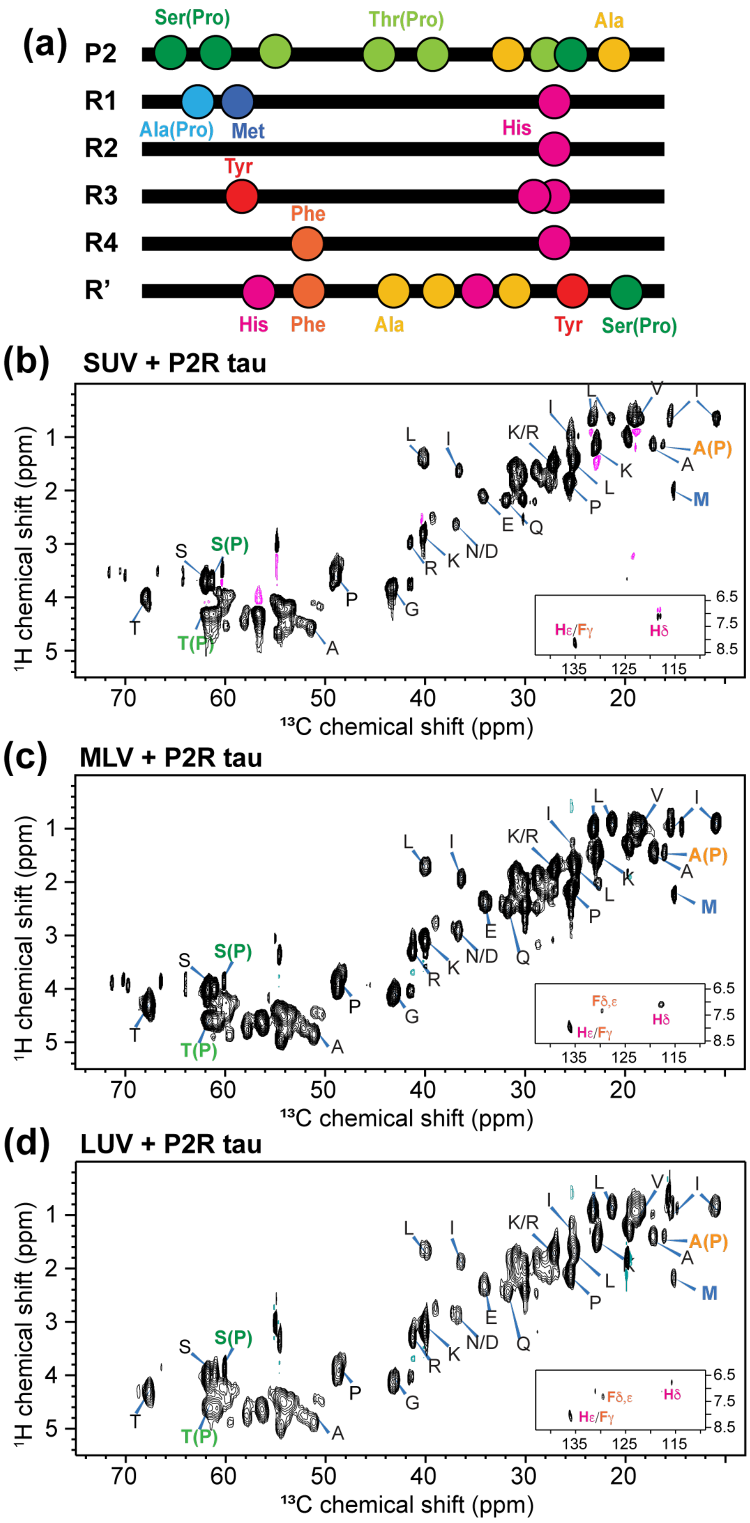

Supplementary Figure 6. **Assignment of highly mobile residues in membrane-bound tau using 2D ^{1}H-^{13}C INEPT spectra.** (**a**) Schematic diagram of the P2R tau amino acid sequence, showing the numbers and distributions of several residue types, including Pro-preceding residues Ser(P), Thr(P), Ala(P), and Ala, Met, Tyr, Phe, and His. (**b**) 2D INEPT spectrum of SUV-bound P2R tau. (**c**) 2D INEPT spectrum of MLV-bound P2R tau. (**d**) 2D INEPT spectrum of LUV-bound

P2R tau. In all three spectra, strong Met, Ala(P), Ser(P), and Thr(P) signals are observed, suggesting that the P2 and R1 domains are flexible. Few or no Phe and Tyr signals are observed, suggesting that the R3-R4-R' domains are rigid or semi-rigid.

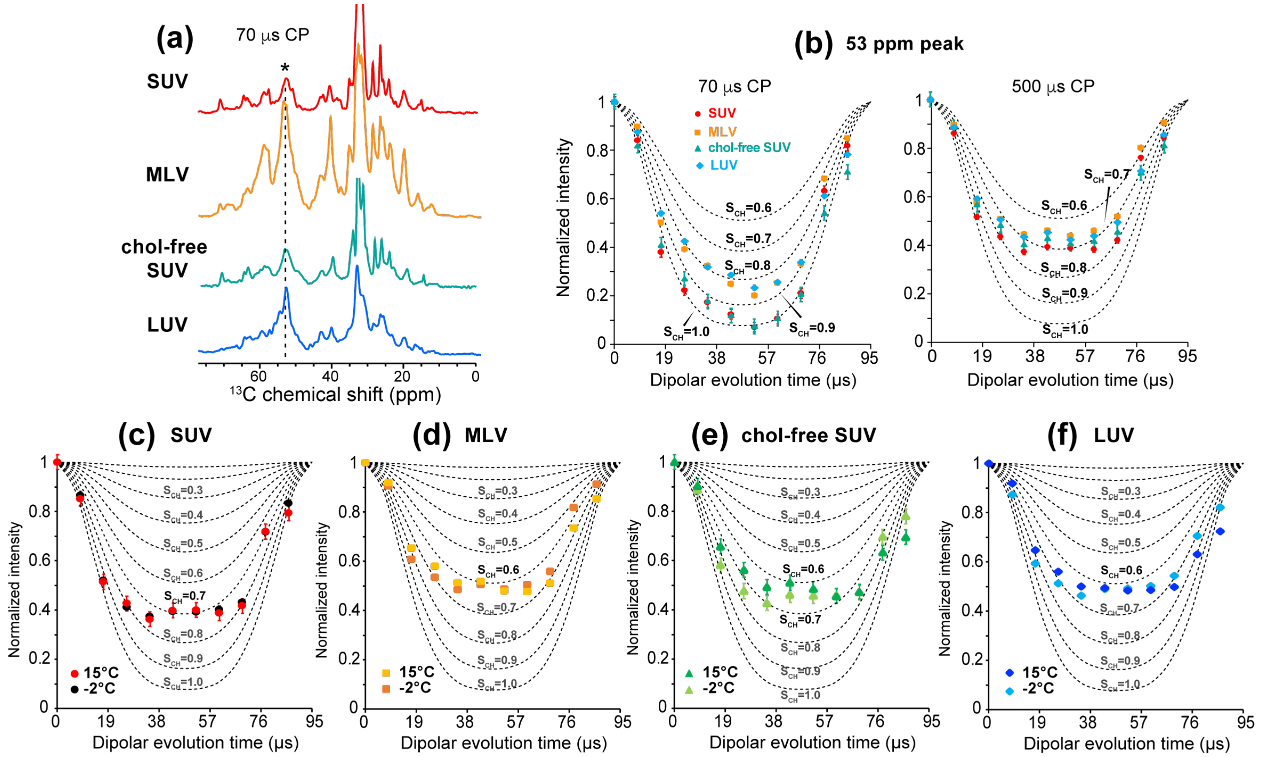

Supplementary Figure 7. ^{13}C-^{1}H dipolar couplings show that the membrane tau mobility is sensitive to the membrane condition but insensitive to temperature. (**a**) ^{13}C cross section from the 2D C-H dipolar-doubled DIPSHIFT spectra of the four membrane-bound P2R tau samples. The spectra were measured with a ^{1}H-^{13}C CP contact time of 70 µs and a constant dipolar evolution time of one rotor period. (**b**) Dipolar-doubled C-H DIPSHIFT curves of the four membrane tau samples measured at -2°C. The integrated intensities of the 53-ppm Cα peak for Leu, Asp, Asn, Lys, Gln and Glu residues are shown. These are overlaid with simulated curves (dotted lines) for varying C-H order parameters (S_{CH}). Left: DIPSHIFT data measured with 70 µs CP to preferentially detect highly rigid residues. Right: DIPSHIFT data measured with 500 µs CP to detect both rigid and semi-rigid residues. (**c-f**) Comparison of the 500 µs CP-DIPSHIFT data of the four membrane tau samples. Each sample was measured at 15˚C and -2˚C to compare the tau mobility. (**c**) SUV-bound tau. (**d**) MLV-bound tau. (**e**) Chol-free SUV-bound tau. (**f**) LUV-bound tau. Plotted data represent the integrated Cα intensities between 50 and 63 ppm. The dephasing curves are similar between -2˚C and 15˚C for each sample, indicating that the protein dynamics is relatively insensitive to temperature. Among the four samples, the SUV-bound tau has the largest order parameter.
