# Supplementary material for: Membrane-induced tau amyloid fibrils
Source: Commun Biol. 2023 Apr 28;6:467. doi: 10.1038/s42003-023-04847-6 (PMC10147698; doi:10.1038/s42003-023-04847-6)
Supplement: Supplementary file 3 — Reporting Summary [file 42003_2023_4847_MOESM3_ESM.pdf]

nature portfolio

Corresponding author(s): Mei Hong

Last updated by author(s): April 4, 2023

Reporting Summary

Nature Portfolio wishes to improve the reproducibility of the work that we publish. This form provides structure for consistency and transparency in reporting. For further information on Nature Portfolio policies, see our Editorial Policies and the Editorial Policy Checklist.

Statistics

For all statistical analyses, confirm that the following items are present in the figure legend, table legend, main text, or Methods section.

n/a	Confirmed	
☐	☒	The exact sample size (*n*) for each experimental group/condition, given as a discrete number and unit of measurement
☐	☒	A statement on whether measurements were taken from distinct samples or whether the same sample was measured repeatedly
☒	☐	The statistical test(s) used AND whether they are one- or two-sided<br>*Only common tests should be described solely by name; describe more complex techniques in the Methods section.*
☒	☐	A description of all covariates tested
☒	☐	A description of any assumptions or corrections, such as tests of normality and adjustment for multiple comparisons
☐	☒	A full description of the statistical parameters including central tendency (e.g. means) or other basic estimates (e.g. regression coefficient) AND variation (e.g. standard deviation) or associated estimates of uncertainty (e.g. confidence intervals)
☒	☐	For null hypothesis testing, the test statistic (e.g. *F*, *t*, *r*) with confidence intervals, effect sizes, degrees of freedom and *P* value noted<br>*Give P values as exact values whenever suitable.*
☒	☐	For Bayesian analysis, information on the choice of priors and Markov chain Monte Carlo settings
☒	☐	For hierarchical and complex designs, identification of the appropriate level for tests and full reporting of outcomes
☒	☐	Estimates of effect sizes (e.g. Cohen's *d*, Pearson's *r*), indicating how they were calculated

Our web collection on statistics for biologists contains articles on many of the points above.

Software and code

Policy information about availability of computer code

Data collection	NMR data acquisition: Bruker Topspin 3.2 & 3.5.
Data analysis	NMR data processing: Bruker Topspin 4.1, 3.2 & 3.5.<br>EM Image analysis: ImageJ 1.53a<br>DIPSHIFT Analysis: Microsoft Excel<br>NMR spectral assignment: CCPNMR 2.5.2<br>Molecular Model: PyMOL

For manuscripts utilizing custom algorithms or software that are central to the research but not yet described in published literature, software must be made available to editors and reviewers. We strongly encourage code deposition in a community repository (e.g. GitHub). See the Nature Portfolio guidelines for submitting code & software for further information.

Data

Policy information about availability of data

All manuscripts must include a data availability statement. This statement should provide the following information, where applicable:

- Accession codes, unique identifiers, or web links for publicly available datasets
- A description of any restrictions on data availability
- For clinical datasets or third party data, please ensure that the statement adheres to our policy

Data are available from M.H. upon reasonable request.

Field-specific reporting

Please select the one below that is the best fit for your research. If you are not sure, read the appropriate sections before making your selection.

☒ Life sciences ☐ Behavioural & social sciences ☐ Ecological, evolutionary & environmental sciences

For a reference copy of the document with all sections, see nature.com/documents/nr-reporting-summary-flat.pdf

Life sciences study design

All studies must disclose on these points even when the disclosure is negative.

Sample size	Fibril widths from TEM data: all particles in multiple images were sampled. This is sufficient for the qualitative conclusions made in this study; Membrane vesicle diameters from TEM images: all vesicles in multiple images were sampled. This is sufficient for the qualitative conclusions made in this study.
Data exclusions	No data were excluded.
Replication	Biochemical characterization (sedimentation assay, TEM) was performed two times prior to preparing NMR samples.<br>Each NMR sample was made once and the NMR spectra were measured over the course of a few months on the same set of samples. Each NMR samples contained >10^16 molecules; thus the NMR spectra report the average over all molecules in the sample. A large number of scans were obtained for each spectrum to obtain sufficient signal-to-noise ratios. Sample size was chosen by maximizing the amount of material that could be fit into the SSNMR rotor.
Randomization	Randomization was not relevant for this study, as samples consist of >10^16 randomly distributed molecules and signal averaging is the physical equivalent to randomization.
Blinding	Blinding was not relevant for this study as no human subjects were studied. Physical data were processed exactly as described and no data were omitted from analysis; physical data is not biased by researcher input.

Reporting for specific materials, systems and methods

We require information from authors about some types of materials, experimental systems and methods used in many studies. Here, indicate whether each material, system or method listed is relevant to your study. If you are not sure if a list item applies to your research, read the appropriate section before selecting a response.

Materials & experimental systems

n/a	Involved in the study	
☒	☐	Antibodies
☒	☐	Eukaryotic cell lines
☒	☐	Palaeontology and archaeology
☒	☐	Animals and other organisms
☒	☐	Human research participants
☒	☐	Clinical data
☒	☐	Dual use research of concern

Methods

n/a	Involved in the study	
☒	☐	ChIP-seq
☒	☐	Flow cytometry
☒	☐	MRI-based neuroimaging
